# All trans-retinoic acid protects against acute ischemic stroke by modulating neutrophil functions through STAT1 signaling

**DOI:** 10.1186/s12974-019-1557-6

**Published:** 2019-08-31

**Authors:** Wei Cai, Julie Wang, Mengyan Hu, Xiao Chen, Zhengqi Lu, Joseph A. Bellanti, Song Guo Zheng

**Affiliations:** 1 Center of Clinical Immunology, Center for Mental and Neurological Disorders and Diseases, the Third Affiliated Hospital of Sun Yat-sen University, Guangzhou, People’s Republic of China; 2 Department of Neurology, Center for Mental and Neurological Disorders and Diseases, the Third Affiliated Hospital of Sun Yat-sen University, 600 Tianhe Road, Guangzhou, 510630 Guangdong China; 3 Department of Internal Medicine, Ohio State University College of Medicine and Wexner Medical Center, Columbus, OH 43201 USA; 4 Division of Hematology and Oncology, Department of Medicine, Medical College of Wisconsin, Milwaukee, WI USA; 5 Department of Pediatrics and Microbiology-Immunology, Georgetown University Medical Center, Washington, DC 20057 USA

**Keywords:** Cerebral ischemia, Neutrophil, Neural inflammation, All trans-retinoic acid, Neuroprotection

## Abstract

**Background and purpose:**

Regulation of neural inflammation is considered as a vital therapeutic target in ischemic stroke. All-trans retinoic acid (atRA), a potent immune modulator, has raised interest in the field of stroke therapy. However, the immunological mechanisms for atRA-mediated neuroprotection remain elusive. The current study evaluated the impact of atRA on post-stroke neural inflammation and elucidated the mechanisms involved in the regulation of related neutrophil functions.

**Methods:**

atRA was prophylactically administered to mice 1 day before transient middle cerebral artery occlusion (tMCAO, 1 h) and repeated daily immediately after reperfusion for 3 days. Stroke outcomes, neutrophil polarization, and formation of neutrophil extracellular traps (NETs) in the stroke lesion were assessed. Neutrophil depletion was induced with anti-Ly6G antibodies. Primary neutrophil cultures were used to explore the mechanisms of atRA treatment.

**Results:**

Prophylactic atRA treatment reduced infarct volumes and neurological deficits at 1 day after tMCAO. Post-stroke neural inflammation was attenuated and neutrophil accumulation in lesion was downregulated. atRA treatment skewed neutrophil toward N2 phenotype which facilitated its clearance by macrophage and inhibited NETs formation. The functions of neutrophil were indispensable in the protective effects of atRA and were associated with suppression to STAT1 signaling by atRA. Administration of atRA after stroke still provided efficient protection to cerebral ischemia.

**Conclusion:**

atRA displays potent therapeutic efficacy in ischemic stroke by attenuating neural inflammation. Treatment of atRA impeded neutrophil accumulation, favored N2 polarization, and forbade NETs formation in ischemic lesion. STAT1 signaling played a decisive role in the mechanisms of atRA-afforded regulation to neutrophil.

**Electronic supplementary material:**

The online version of this article (10.1186/s12974-019-1557-6) contains supplementary material, which is available to authorized users.

## Background

Neural immune responses play critical roles in ischemic stroke through mechanisms which paradoxically mediate brain injury or recovery. Neutrophil is among the forerunners to the ischemic lesion. After summoned by neutrophil-attracting chemokines, neutrophil exerts elaborate functions within stroke lesion. Similar to microglia/macrophage, neutrophil displays heterogeneous phenotypes. It was demonstrated that N2 neutrophil facilitated phagocytic activities of microglia/macrophage which benefited inflammatory resolution after stroke, while the N1 phenotype aggravated neural inflammation [1]. In addition, formation of neutrophil extracellular traps (NETs), the extracellular chromatin-containing products of neutrophils, has been shown to exacerbate neuronal injury after stroke [2]. Modulation of neutrophil activities, therefore, represents a promising therapeutic strategy for stroke therapy.

All-trans retinoic acid (atRA) is the ligand of retinoic acid receptors (RARs) and displays potent immunoregulatory effects on autoimmune diseases [3–8]. Recent studies have documented that prophylactic administration of atRA reduced brain injury in stroke as neuronal viability and blood-brain barrier (BBB) integrity were improved and glia cell activation was controlled [9–11]. Nevertheless, the underlying mechanisms of the protection offered by atRA remain to be elusive.

The current study explored the impact of prophylactic atRA treatment on post-stroke neural inflammation and the underlying mechanisms. Our results showed that atRA decreased neutrophil accumulation, directed neutrophil polarization toward N2 phenotype, and reduced NETs formation in stroke lesion. Mechanistically, atRA suppressed signal transducers and activators of transcription 1 (STAT1) signaling through enhancing the expression of suppressor of cytokine signaling-1 (SOCS1). Furthermore, we evaluated therapeutic efficacy of post-stroke atRA treatment. Reduced infarct lesion and improved neurological functions were documented.

## Methods

Our manuscript completely adheres to the AHA Journals’ implementation of the Transparency and Openness Promotion (TOP) Guidelines.

### Animals

Animals in the current study were purchased from Guangdong Medical Laboratory Animal Center (Guangzhou, China), and housed in the animal facility in Sun Yat-sen University. The animal facility was humidity- and temperature-controlled with a 12-h light-dark cycle. Animals were housed in the animal facility for at least 1 week before induction of ischemic stroke. Food and water were freely accessible. Experimental procedures in the current study were in compliance with the National Institutes of Health’s Guide for the Care and Use of Laboratory Animals. Experimental protocols were approved by the Animal Care and Use Committee of Sun Yat-sen University following the Guide for the Care and Use of Laboratory Animals and Stroke Treatment. A total of 207 male and six female C57BL/6 mice (8- to 12-week-old, weight 18–25 g) were used in the study (Additional file 1: Table S1).

### Ischemic stroke models

Focal ischemic stroke model was induced in mice with transient middle cerebral artery occlusion (tMCAO) as described previously [12]. Briefly, mice were anesthetized with 1.5% isoflurane in air under spontaneous breathing. A filament was inserted into external carotid artery (ECA) and directed to the middle cerebral artery (MCA) through the internal carotid artery (ICA). Filament insertion into the ICA was maintained for 60 min followed by reperfusion. Core body temperature was maintained with a heating pad. The cerebral blood flow (CBF) during the surgery was measured by laser Doppler flowmetry. Mice with less than 70% reduction of blood flow in the ischemic core or that died during surgery were excluded from further analyses. A total of 174 mice were subjected to tMCAO. Ten mice were excluded from the study including nine which died after surgery and one with neurological deficit score < 1. Mortality of tMCAO mice was 5.2% (Additional file 1: Table S1). A total of six mice were subjected to Sham operation with procedure same as tMCAO except for no filament insertion.

### Drug administration

All-trans retinoic acid (Sigma, St. Louis, MO, USA. R2625) was dissolved in dimethyl sulfoxide (DMSO) and further diluted in PBS. At 1 day before tMCAO or Sham operation, atRA was administered (1 mg/kg, minimum effective dose for stroke treatment identified by Sato. et al. [13]) intraperitoneally (i.p.) to mice and the injection was repeated daily consecutively starting immediately after reperfusion until time point of sacrifice. For post-stroke atRA treatment, atRA was administered (1 mg/kg) i.p. to mice for one dose at 2 h after reperfusion. Animals in the PBS-treated group received an equivalent volume of PBS i.p. injection. All outcome endpoints were measured by investigators blinded to experimental group assignments. For neutrophil depletion, 50 μg of anti-Ly6G antibodies (Thermo Fisher, Carlsbad, CA, USA) was intravenously injected to mice at 2 days before tMCAO.

### Infarct volume analysis

The volumes of brain infarct were assessed with 2,3,5-Triphenyltetrazolium chloride (TTC) (Sigma, St. Louis, MO, USA. T8877) staining. A series of six sections in the middle cerebral artery territory was obtained from each mouse brain and stained with TTC. TTC was dissolved in sterile saline to a concentration of 4% and was used to stain the freshly cut brain coronal slices (1 mm thick) followed by fixation of the slices with PBS containing 4% paraformaldehyde (PFA, Sigma, St. Louis, MO, USA). The area of infarct lesion for each section was determined as the difference between the TTC-positive area of contralateral hemisphere and ipsilateral hemisphere. Brain infarct was determined by multiplying the mean area of tissue loss by the distances between the two adjacent stained brain slices (1 mm).

### Neurological deficit scoring

Neurological deficit scores were assessed immediately after ischemia-reperfusion (0 day) and 1 day after tMCAO. Neurological deficit scores were determined on a 0–4 scale: 0 = no apparent deficit, 1 = weakness in the ipsilateral forelimb (right), 2 = circulating to the ipsilateral side (right), 3 = body unbalance and trunk incline to ipsilateral, and 4 = no spontaneous motor activity or death [14].

### Immunofluorescence staining and cell quantification

Animals were euthanized and perfused with PBS followed by 4% PFA. Brains were removed and cut into 25 μm frozen cryosections using a microtome. Brain sections were incubated with primary antibodies at 4 °C overnight. After washing with PBS, sections were incubated with secondary antibodies for 1 h at room temperature. Sections were then washed and mounted with DAPI Fluoromount-G (Thermo Fisher, Carlsbad, CA, USA). The following primary antibodies were used: rabbit anti-NeuN (Clone: A50, 1:500, EMD Millipore, Billerica, MA), rabbit anti-Iba1 (Clone: NCNP24, 1:1000, Wako Pure Chemical Industries, Osaka, Japan), rat anti-Ly6G (Clone: 1A8-Ly6g, 1:500, Thermo Fisher, Carlsbad, CA, USA), and rabbit anti-citrullinated histone 3 (Multi-clonal, 1:500 Abcam, Cambridge, USA). The following secondary antibodies were applied: anti-rabbit secondary antibody conjugated with Cy3 (1:1000, Jackson ImmunoResearch Laboratories, West Grove, PA, USA), anti-rabbit secondary antibody conjugated with Alexa Fluor 488 (1:1000, Jackson ImmunoResearch Laboratories, West Grove, PA, USA), and anti-rat secondary antibody conjugated with Alexa Fluor 488 (1:1000, Jackson ImmunoResearch Laboratories, West Grove, PA, USA). For neuronal apoptosis analysis, terminal deoxynucleotidyl transferase dUTP nick end labeling (TUNEL) was processed after NeuN labeling according to instructions from the manufacturer (Thermo Fisher, Carlsbad, CA, USA). Immunopositive cell quantification and area analysis were performed with the software of ImageJ (National Institutes of Health) by an investigator who was blinded to the experimental design. In quantification of cell in stroke penumbra, the stroke core was identified as the region in which the majority of DAPI-stained nuclei were shrunken, and the stroke penumbra was defined as the region of generally morphologically normal cells, approximately 450–500 μm wide, surrounding the stroke core [15]. To confirm the RARs expression in primary cultured neutrophils, cells were stained with rabbit anti-RARα (Clone: H1920, 1:500, Abcam, Cambridge, USA), rabbit anti-RARβ (Multi-clonal, 1:500, Abcam, Cambridge, USA), and rabbit anti-RARγ (Multi-clonal, 1:500, Abcam, Cambridge, USA). Phalloidin-Alexa Fluor 488 (Thermo Fisher, Carlsbad, CA, USA) was used to label actin in neutrophils.

### Quantitative polymerase chain reaction

Total RNA was isolated from brains, magnetically selected cells or primary cultured neutrophil using the RNeasy Mini Kit (Qiagen, Valencia, CA, USA) according to the manufacturer’s instructions; 1 μg RNA (OD260 nm/OD280 nm = 1.8–2.2) was used to synthesize the first strand of cDNA using the PrimeScript RT reagent Kit (Takara Bio Inc., Shiga, Japan). Quantitative polymerase chain reaction (qPCR) was performed on a 7500 fast (ABI) RT-PCR machine using SYBR Premix Ex Taq (Takara Bio Inc., Shiga, Japan). Primers used in the study were listed in Additional file 1: Table S2. For qPCR, the following program was performed: 95 °C 30 s (pre-denaturation); 95 °C 5 s and 60 °C 34 s for 40 cycles (amplification); 95 °C 15 s, 60 °C 1 min and 95 °C 15 s (melt curve). Double delta CT log2 were calculated and the data presented as fold change normalized to PBS-treated contralateral brain, neutrophil from PBS-treated mice, or DMSO-treated neutrophil. Glyceraldehyde-3-phosphate dehydrogenase (GAPDH) was used as a normalizer housekeeping gene.

### Flow cytometric analysis

Single brain cells were prepared for flow cytometric analysis (FACS). Briefly, brains were dissected and ipsilateral hemispheres were collected. Each hemisphere was subjected to digestion with 0.25% trypsin-EDTA (Thermo Fisher, Carlsbad, CA, USA) at 37 °C for 25 min. Brain tissue was then pressed through a cell strainer (70 μm). Brain cells were separated from myelin debris by centrifugation in 30%/70% Percoll solution (GE Healthcare Biosciences AB, Uppsala, Sweden). Brain cells at the interface were collected, washed with HBSS, and subjected to further staining. The following antibodies were used: CD45-PerCp/Cy5.5 (clone: 30F11, 1:400, Biolegend, San Diego, CA), CD11b-PE (clone: M1/70, 1:400, Biolegend, San Diego, CA), Ly6G-APC/Cy7 (clone: 1A8, 1:400, Biolegend, San Diego, CA), F4/80-BV421 (clone: BM8, 1:400, Biolegend, San Diego, CA), CD206-Alexa Fluor 647 (clone: 19.2, 1:200, BD bioscience, San Diego, CA), Ym1/2-Alexa Fluor 488 (clone: EPR15263, 1:100, Abcam, Cambridge, USA), Arg1-APC (Polyclonal, 1:200, R&D Systems, Minneapolis, MN), pSTAT1-PE CF 594 (Clone: 4a, 1:100, BD bioscience, San Diego, CA), rabbit anti mouse SOCS1 (Polyclonal, Abcam, Cambridge, USA, 1:100), and IFNγ-Alexa Fluor 488 (Clone: XMG1.2, 1:100, Thermo Fisher, Carlsbad, CA, USA). Anti-rabbit-Alexa fluor 488 was used as a secondary antibody after incubation with rabbit anti-mouse SOCS1. FACS was performed using a fluorescence-activated cell sorter flow cytometer (BD bioscience, San Diego, CA), and data were analyzed using FlowJo X 10.0.7r2 software. Appropriated isotype controls were stained following the manufacturer’s instruction (Thermo Fisher, Carlsbad, CA, USA). Fluorchrome compensation was performed with single-stained OneComp eBeads (Thermo Fisher, Carlsbad, CA, USA). As for data presentation, when cells could be divided into negative or positive populations, percentage of cells was calculated. When expression of coordinated marker was consecutive and population separation was obscure, data were presented as mean fluorescence intensity (MFI).

### Magnetic sorting of neutrophils

Single brain cells were prepared as described previously. Cells from each hemisphere were stained with Biotin-anti-Ly6G (Clone: 1A8, Biolegend, San Diego, CA) (1 μg antibodies per 10^6^ cells). Cells were washed with HBSS and further stained with anti-Biotin microbeads (Miltenyi Biotec, Bergisch Gladbach, Germany) (1 μg antibodies per 10^6^ cells). After two washes with HBSS, cells were subjected to a positive selection program using autoMACS (Miltenyi Biotec, Bergisch Gladbach, Germany). Purity of neutrophil extracted with the kit was ~ 92.1% (average from three tests). Cells were washed and then proceeded to RT-PCR and qPCR.

### Primary neutrophil-enriched cultures and extracellular traps induction

Primary neutrophil-enriched cultures were prepared from bone marrow of 8- to 10-week-old healthy C57BL/6 male mice using EasySep Mouse Neutrophil Enrichment Kit (StemCell Technologies, Vancouver, CA, 9762) according to manufacturer’s instructions. Purity of neutrophil extracted with the kit was ~ 88.7% (average from three tests). For induction of neutrophil extracellular traps (NETs), neutrophils were treated with 20 nM of phorbol 12-myristate 13-acetate (PMA, Sigma, St. Louis, MO, USA) in serum-free cultured medium (RMPI1640) for 3 h. After stimulation, normal culture medium was re-applied to neutrophils and condition medium (CM) was collected 24 h later. In specific experiment, anisomycin (25 μg/ml, Sigma, St. Louis, MO, USA) was treated to neutrophil so as to over-activate STAT1 signaling and confront the function of SOCS1.

### Primary cortical neuronal cultures and oxygen glucose deprivation

Primary cortical neuronal cultures were prepared from E16-18 embryos of C57BL/6 mice as previously described [12]. The density of neurons seeded for oxygen glucose deprivation (OGD) studies was 3.5 × 10^5^ per well in 24-well plate. Neuronal ischemia was induced with OGD. Briefly, neuron culture medium (Neural basal + B27 + 2% glutamate) was retreated and replaced by Earle’s balance salt solution (EBSS). Neurons were then incubated in 95% N2 + 5% CO2 for 6 h. After OGD, normal neuron medium was re-applied for reperfusion and returned to regular atmospheric oxygen level in normal incubator. At 24 h after OGD, viability of neurons was assessed with immunofluorescence staining of NeuN (1:500, EMD Millipore, Billerica, MA). For calculation of lived neurons, we randomly selected four4 fields with microscope and counted the cells in the fields with ImageJ. Data were presented as NeuN^+^ cells/mm^2^.

### Western blot

Protein isolation from cultured neutrophil was performed as previously described [16]. Western blots were performed using the standard SDS-polyacrylamide gel electrophoresis method and enhanced chemiluminescence detection reagents (GE Healthcare Biosciences AB, Uppsala, Sweden). Antibodies against phosphorylated signal transducer and activator of transcription 1 (pSTAT1) (Multi-clonal, Abcam, Cambridge, USA), STAT1 (Multi-clonal, Cell signaling technology, Beverly, MA), SOCS1 (Multi-clonal, Abcam, Cambridge, USA), and GAPDH (Cell signaling technology, Beverly, MA) were used. Immunoreactivity was semi-quantitatively measured by gel densitometric scanning and analyzed by the MCID image analysis system (Imaging Research, Inc.).

### Chromatin immunoprecipitation

A total of 2 million cells per sample of primary cultured neutrophils were processed for chromatin immunoprecipitation (ChIP) analysis. ChIP experiments were performed using Thermo Scientific Pierce Chromatin Prep Module (Thermo Fisher, Carlsbad, CA, USA, 26158) and Pierce Agarose ChIP Kit (Thermo Fisher, Carlsbad, CA, USA, 26156) according to manufacturer’s instructions as previously described [17]. Rabbit anti-RARα (Abcam, Cambridge, USA), rabbit anti-RARβ (Abcam, Cambridge, USA), or rabbit anti-RARγ (Abcam, Cambridge, USA) (5 μg per sample) antibodies were used for chromatin extraction. In each ChIP experiment, 3 μl of template was applied to 25 μl PCR system (Vazyme, Nanjing, China. P505-d1) for expansion. The primers for SOCS1 were used (Additional file 1: Table S2) and the following PCR program were used: 95 °C 5 min, 94 °C 30 s, 57 °C 30 s, 72 °C 55 s; for 50 cycles.

### Statistical analysis

All results were presented as mean ± standard error of the mean (SEM). The differences in the means among multiple groups were analyzed using one- or two-way analysis of variance (ANOVA). When ANOVA showed significant differences, pair-wise comparisons between means were tested by Dunnett’s test. The Student’s *t* test was used for two-group comparisons. In all analysis, *P* < 0.05 was considered statistically significant.

## Results

### All-trans retinoic acid protected against ischemic stroke

**Fig. 1:**
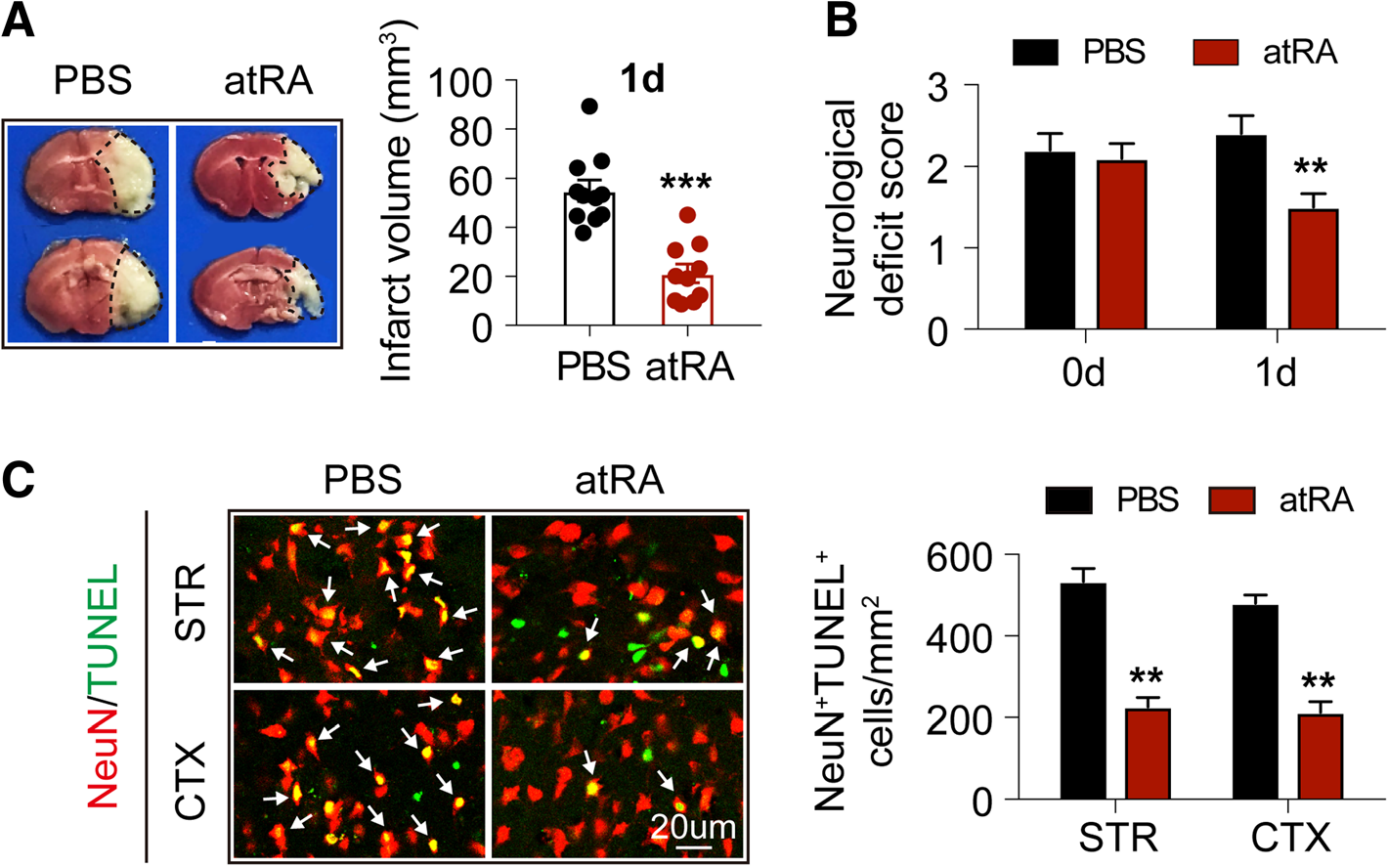
Prophylactic atRA treatment protected against acute ischemic stroke. Male C57BL/6 mice were treated with atRA (1 mg/kg, i.p.) or PBS 24 h before 60 min of cerebral ischemia. The treatment was repeated immediately after reperfusion. Mice were sacrificed at 1 day after tMCAO. **a** Infarct volume of mice was quantified with TTC (red)-stained coronal sections (*N* = 11 mice for PBS-treated group and *N* = 10 mice for atRA-treated group). ***P ≤ 0.001, versus PBS-treated group in *t* test. **b** Neurological deficit score was assessed right after reperfusion and 1d after tMCAO (*N* = 11 mice for PBS-treated group and *N* = 10 mice for atRA-treated group). **P ≤ 0.01, versus PBS-treated group in two-way ANOVA. **c** *Left*: Representative images showing TUNEL (green) co-labeling with NeuN (red) in infarct penumbra at 1d after tMCAO. *Right:* Quantification of the number of NeuN^+^TUNEL^+^ neurons (yellow, emphasized with white arrows) in stroke penumbra of striatum (STR) and cortex (CTX). *N* = 6 mice per group. **P ≤ 0.01, versus PBS-treated group in *t* test

Male C57BL/6 wild-type (WT) mice were treated with atRA (1 mg/kg, i.p.) or PBS at 24 h before tMCAO and immediately after reperfusion. In accordance to previous findings, prophylactic atRA treatment improved stroke outcomes as atRA pre-treated mice exhibited significantly reduced infarct volumes (Fig. 1a) and lower neurological deficit scores (Fig. 1b). For analysis of neuronal death, terminal deoxynucleotidyl transferase dUTP nick end labeling (TUNEL, green) was co-stained with neuron-specific nuclear-binding protein (NeuN, red) in brain slices. We found that mice with prophylactic atRA treatment had less dead neurons ((NeuN^+^TUNEL^+^, emphasized with white arrows) in stroke penumbra in both striatum (STR) and cortex (CTX) (Fig. 1c) at 1 day after cerebral ischemia when compared to PBS-treated control. No TUNEL signal was detected in the contralateral brains of PBS- or atRA pre-treated mice (Additional file 1: Figure S1A).

### Prophylactic atRA treatment attenuated post-stroke neural inflammation and reduced neutrophil accumulation in stroke lesion

**Fig. 2:**
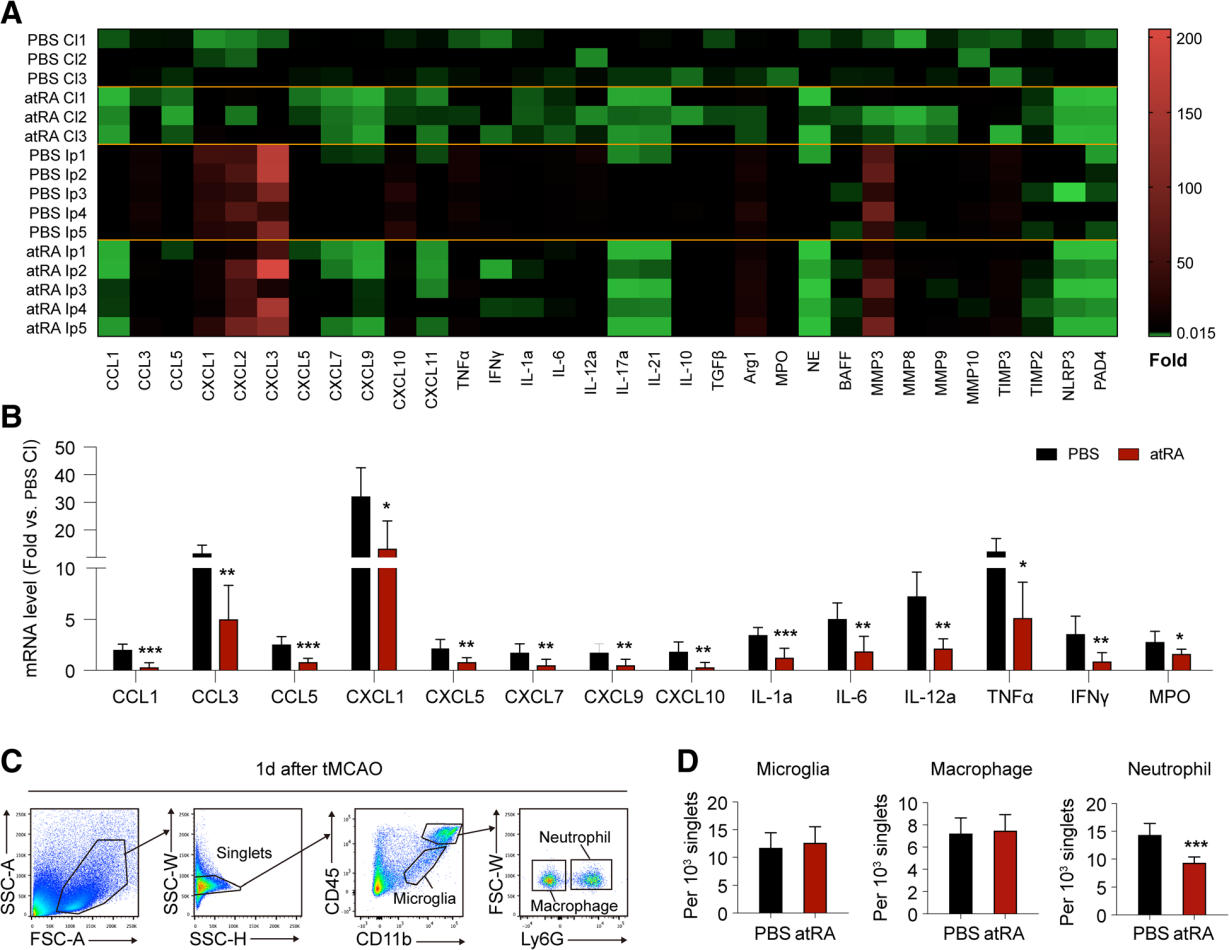
Prophylactic atRA treatment attenuated post-stroke neural inflammation. Post-stroke neural inflammation at acute phase after cerebral ischemia was analyzed with qPCR (**a**, **b**) and flow cytometry (**c**, **d**) at 1 day after tMCAO. **a** Heat map of the mRNA expression of inflammatory mediators in contralateral brain (Cl) and ipsilateral hemisphere (Ip). In the heat map, data are displayed as fold change to Cl of PBS-treated group. Black, expression un-altered, fold change = 1; Red, expression up-regulated, fold change > 1; Green, expression down-regulated, fold change < 1. **b** Quantification of inflammatory markers with significant difference of mRNA expression in the Ip brains between atRA- and PBS-treated mice. Comparison between PBS Cl and atRA Cl groups was displayed in Additional file 1: Figure S1A. Quantification of markers that without significant difference between PBS Ip and atRA Ip groups was displayed in Additional file 1: Figure S1B. mRNA expression was normalized to the level of the Cl brain from PBS-treated mice. *N* = 3 in PBS Cl and atRA Cl groups, *N* = 5 in PBS Ip and atRA Ip groups. *P ≤ 0.05, **P ≤ 0.01, ***P ≤ 0.001, versus PBS Ip in *t* test. **c** Gating strategy for microglia (CD11b^+^CD45^int^), macrophage (CD11b^+^CD45^hi^Ly6G^−^), and neutrophil (CD11b^+^CD45^hi^Ly6G^+^) in flow cytometric analysis. **d** Quantification of microglia, macrophages and neutrophils among single brain cells (singlets). The number in flow panels represents the number of cells per 10^3^ singlets in ipsilateral brain. *N* = 6 mice per group. ***P ≤ 0.001 versus PBS-treated group in *t* test

To evaluate the effects of immune regulation by prophylactic atRA treatment (administering atRA (1 mg/kg, i.p.) at 24 h before tMCAO and immediately after reperfusion), mRNA was isolated from ipsilateral (Ip) or contralateral (Cl) hemisphere at 24 h after tMCAO and expression of inflammatory markers were analyzed with qPCR. Strikingly, expression of multiple inflammatory factors was dramatically downregulated in stroke lesion of atRA pre-treated mice (Fig. 2a, b, Additional file 1: Figure S2). Of particular interest, mRNA expression of neutrophil attracting chemokines significantly decreased in atRA pre-treated group such as chemokine (C-C motif) ligand 5 (CCL5), chemokine (C-X-C motif) ligand 1 (CXCL1), chemokine (C-X-C motif) ligand 5 (CXCL5), and chemokine (C-X-C motif) ligand 7 (CXCL7) (Fig. 2a, b) [18]. We next checked the impact of prophylactic atRA treatment on immune cell infiltration at the acute phase of stroke. Since it is established that neutrophils and macrophages are the main immune cells in the ischemic lesions at 1–2 days after cerebral ischemia, while lymphocytes infiltration predominates after 3 days [19], we focused on the accumulation of macrophage and neutrophil in brain lesion at 1 day after stroke. Quantification of neutrophil and macrophage was assessed with flow cytometry (1 day) (Fig. 2c). Interestingly, we found that prophylactic atRA treatment markedly reduced neutrophil counts in stroke lesion while exerted little impact on macrophage number at 1 day after cerebral ischemia (Fig. 2d). There was no difference in cell count of microglia between the two groups (Fig. 2d). Cell count of microglia (CD11b^+^CD45^int^) in Sham-operated mice between PBS- and atRA-treated group was comparable (Additional file 1: Figure S1D). Few leukocytes (CD45^hi^) were identified in the Sham-operated brains. Nevertheless, cell count of leukocytes in the brain of Sham-operated mice between PBS- and atRA-treated group was in consistence (Additional file 1: Figure S1D). Thus, we infer that the protection offered by atRA in acute ischemic stroke is associated with its modulation to neutrophil. We have demonstrated that neutrophil-attracting chemokines in stroke lesion were downregulated by prophylactic atRA treatment. We next checked if neutrophil clearance was enhanced by administration of atRA.

### Prophylactic atRA treatment directed neutrophil toward N2 phenotype and promoted neutrophil clearance after stroke

**Fig. 3:**
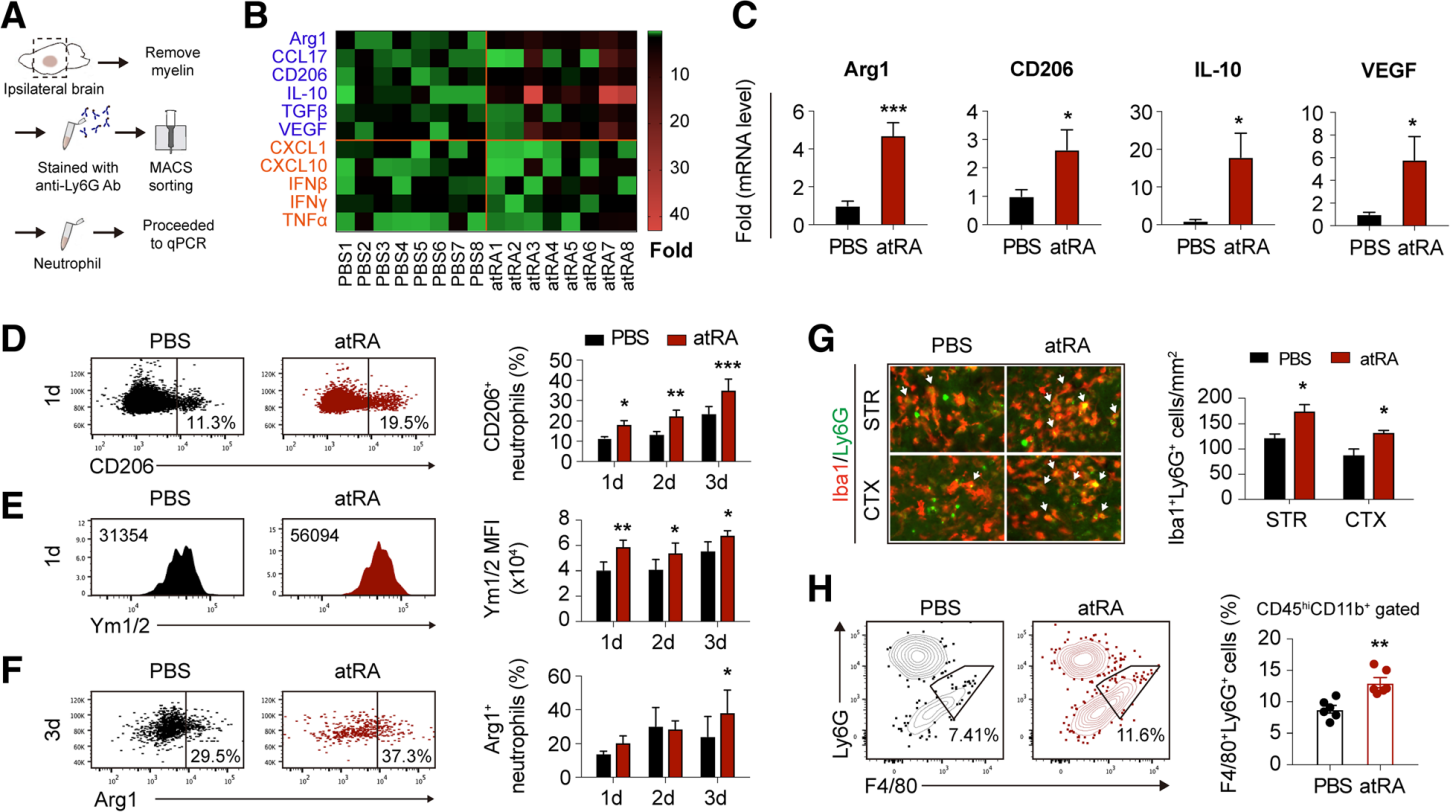
Systemic atRA therapy skewed neutrophil towards N2 phenotype and facilitated neutrophil clearance by microglia / macrophage. **a** Schematic diagram showing that neutrophils in ipsilateral brain were magnetically sorted with anti-Ly6G antibodies and subjected to qPCR. **b** Heat map showing the phenotypic shift of neutrophil in stroke lesion of atRA pre-treated mice. Multiple N1 (orange characters) and N2 (blue characters) markers were analyzed with qPCR. In the heat map, data are displayed as fold change to neutrophils from PBS-treated group. Black, expression un-altered, fold change = 1; Red, expression up-regulated, fold change > 1; Green, expression downregulated, fold change < 1. **c** Quantification of phenotypic markers that showed significant mRNA expression alteration in neutrophil from atRA-treated mice compared with those from PBS-treated mice. Quantification of markers that without significant difference between the two groups were displayed in Additional file 1: Figure S2. *N* = 8 mice per group. *P ≤ 0.05, ***P ≤ 0.001, versus PBS group in *t* test. **d**–**f** Phenotypic shift of neutrophil from brain lesion after atRA treatment was evaluated with flow cytometry at 1–3 days after tMCAO. Representative flow panels and quantification of the percentage of CD206^+^ (**d**) (percentage among CD11b^+^CD45^hi^Ly6G^+^ neutrophil), Ym1/2^+^ (**e**) (mean fluorescence intensity (MFI) among CD11b^+^CD45^hi^Ly6G^+^ neutrophil), and Arg1^+^ (**f**) (percentage among CD11b^+^CD45^hi^Ly6G^+^ neutrophil) of neutrophil were shown. *N* = 4 mice per group. *P ≤ 0.05, **P ≤ 0.01, ***P ≤ 0.001, versus PBS-treated group in two-way ANOVA. **g**–**h** Clearance of neutrophil by microglia / macrophage was assessed with immunofluorescent staining and flow cytometry at 3d after tMCAO. **g** *Left*: Representative images demonstrating neutrophil (Ly6G^+^, green) engulfed by microglia/macrophage (Iba1^+^, red) in infarct penumbra at 3d after tMCAO. The engulfed neutrophil by microglia/macrophage (Ly6G^+^Iba1^+^, yellow) was emphasized with white arrows. *Right*: Quantification of Ly6G^+^Iba1^+^ engulfed neutrophil by microglia/macrophage. *N* = 4 mice per group. *P ≤ 0.05, versus PBS group in *t* test. **h** Neutrophil (CD11b^+^CD45^hi^Ly6G^+^) engulfed by macrophage (CD11b^+^CD45^hi^F4/80^+^) in stroke lesion at 3 days after tMCAO was gated as CD11b^+^CD45^hi^Ly6G^+^F4/80^+^ cells. Representative flow panels and quantification of CD11b^+^CD45^hi^Ly6G^+^ F4/80^+^ engulfed neutrophil among CD11b^+^CD45^hi^ cells were shown. *N* = 6 mice per group. **P ≤ 0.01, versus PBS group in *t-test*. MACS, magnetic cell sorting

Similar to microglia/macrophage, neutrophil can be divided into pro-inflammatory N1 and anti-inflammatory N2 subtypes. N2 neutrophils are inclined to be cleared by microglia/macrophage after cerebral ischemia [1], thus promoting inflammatory resolution. Since neutrophil clearance could be affected by its phenotype, we explored the phenotypic shift of neutrophils in atRA pre-treated mice (administering atRA (1 mg/kg, i.p.) at 24 h before tMCAO and immediately after reperfusion). At 1 day after tMCAO, neutrophils in stroke lesion were isolated with magnetic cell sorting then subjected to qPCR to evaluate the phenotypic profile (Fig. 3a). The mRNA expression of multiple N1 (CXCL1, CXCL10, IFNβ, IFNγ, TNFα) and N2 (Arg1, CCL17, CD206, IL-10, TGFβ, and VEGF) markers [20] were analyzed. Neutrophils from mice pre-treated with atRA expressed higher level of arginase 1 (Arg1), CD206, interleukin 10 (IL-10), and vascular endothelial growth factor (VEGF) (Fig. 3b, c), which revealed enhanced N2 profile of neutrophil. Nevertheless, the expression of N1 markers remained stable after atRA treatment (Fig. 3b, Additional file 1: Figure S3). We further assessed neutrophil polarization in stroke lesion with flow cytometry at 1–3 days after stroke. In atRA pre-treated group, mice were given atRA (1 mg/kg, i.p.) at 24 h before tMCAO and immediately after reperfusion. Injection of atRA was repeated every 24 h until sacrifice. Accordingly, expression of N2 markers including CD206 (Fig. 3d), Ym1/2 [1] (Fig. 3e), and Arg1 (Fig. 3f) significantly increased in neutrophils from the lesion of atRA pre-treated mice. We further evaluated the impact of atRA treatment on neutrophil clearance with immunostaining. As expected, engulfment of neutrophil (Ly6G^+^, green) by microglia/macrophage (Iba1^+^, red) was increased in the stroke penumbra of atRA pre-treated mice in both striatum (STR) and cortex (CTX) (Fig. 3g). No neutrophil was identified in the contralateral brains from both groups (Additional file 1: Figure S1B). Meanwhile, flow cytometric analysis showed higher percentage of CD45^hi^CD11b^+^F4/80^+^Ly6G^+^ cells among CD45^hi^CD11b^+^ cells, which indicated that phagocytosis of neutrophil (CD45^hi^CD11b^+^ Ly6G^+^) by macrophage (CD45^hi^CD11b^+^F4/80^+^) was enhanced (Fig. 3h). Our data demonstrated that atRA directed neutrophil polarization toward beneficial N2 phenotype, and facilitated neutrophil clearance by microglia/macrophage.

### Treatment of atRA inhibited formation of neutrophil extracellular traps

**Fig. 4:**
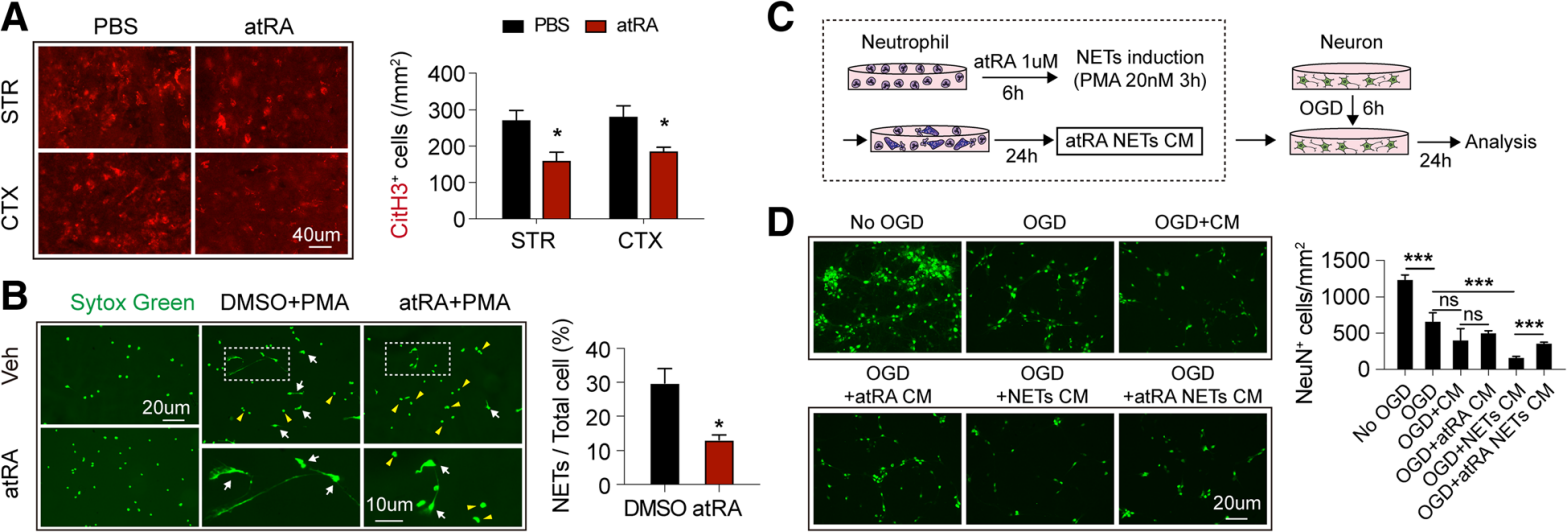
Prophylactic atRA treatment inhibited NETs formation. **a** In situ NETs formation was assessed with immunofluorescent staining of citrullinated histone 3 (CitH3, red) in the stroke lesion at 3 days after tMCAO. Representative images and quantification of NETs were shown. *N* = 4 mice per group. *P ≤ 0.05, versus PBS-treated group in *t* test. **b** Primary culture of bone marrow derived neutrophil was pre-treated with atRA (1 μM for 6 h) then subjected to NETs induction with PMA (20 nM, 3 h). Neutrophil was stained with Sytox green (fluorescent dye of nucleic acids). NETs were identified as the enlarge cloudlike structure (emphasized white arrow) while intact neutrophils were with the morphology of rounded shape (emphasized with yellow arrow head). Representative images and quantification of NETs were presented. Experiments were repeated for three times. *P ≤ 0.05, versus DMSO-treated group in *t* test. **c** Schematic diagram showing the collection process of atRA NETs conditioned medium (CM) and the following treatment to ischemic neurons. Primary cultured neutrophils were first pre-treated with atRA (1 μM for 6 h) then subjected to NETs induction with PMA (20 nM, 3 h). After retreating PMA, NETs forming neutrophils were cultured with normal neutrophil medium for another 24 h and CM was collected. Primary cultured neurons were subjected to 6 h of oxygen glucose deprivation (OGD) followed by reperfusion with normal neuron medium. CM from atRA pre-treated NETs forming neutrophil (atRA NETs CM) was applied to the re-perfused neurons (CM: neuron medium = 1:1) for another 24 h. Viability of the ischemic neurons was assessed with immunofluorescent staining of NeuN. **d** Representative images of neuronal viability and quantification of lived neurons (NeuN^+^ green) were displayed. Experiments were repeated for three times. ***P ≤ 0.001 in one-way ANOVA

Formation of neutrophil extracellular traps (NETs) represents a detrimental aspect of neutrophil function in aseptic inflammation [21]. NETs is a programmed cell death process [22] which is accompanied with release of DNA, free radicals, superoxide, and numerous enzymes [23]. Since atRA protected against ischemic stroke and could modulate neutrophil functions, we sought to evaluate the impact of atRA treatment on NETs formation. Citrullination of histone has been shown to be indispensable for NETs formation [21]. Therefore, citrullinated Histone-3 (CitH3) was used as a marker of NETs formation. As detected by immunostaining, atRA pre-treated mice displayed significantly less CitH3 (red) in stroke penumbra in both striatum and cortex (Fig. 4a). No signal of CitH3 was detected in the contralateral brains of PBS- or atRA pre-treated mice (Additional file 1: Figure S1C). In an in vitro experiment, we pre-treated neutrophil with atRA (1 μM) or DMSO for 6 h, following by NETs induction with phorbol 12-myristate 13-acetate (PMA, 20 nM) for 3 h. As detected with Sytox green, a fluorescent dye for nucleic acids, NETs were identified as the enlarge cloudlike structure (white arrow) while intact neutrophils were with the morphology of rounded shape (yellow arrow head). We found that NETs were less detected in atRA pre-treated neutrophils (Fig. 4b). We next analyzed the influence of NETs on the viability of ischemic neurons. Conditioned medium (CM) was collected after NETs induction with or without atRA and was then treated to neurons subjected to oxygen glucose deprivation (OGD). Neuronal viability was assessed with immunostaining of NeuN after 24 h of CM treatment (Fig. 4c). CM of integral (CM) or atRA pre-treated neutrophils (atRA CM) did not cause further decline of neuron counts after OGD (OGD). In contrast, CM from NETs forming neutrophils (NETs CM) showed significant toxicity to OGD neurons (OGD). Nevertheless, CM of atRA pre-treated NETs forming neutrophils (atRA NETs CM) was less harmful to the ischemic neurons (OGD) (Fig. 4d). Thus, atRA impeded the formation of NETs and attenuated the detrimental impact of NETs on ischemic neurons, which benefited survival of neuron in stroke. The current grouping could not totally discard the effect of atRA left in the CM on neuronal survival. However, neuronal viability showed no difference between OGD + atRA CM and OGD + CM, which indicated that atRA left in the CM has little effect on neuronal survival (Fig. 4d).

### All-trans retinoic acid failed to offer further protection to mice after neutrophil depletion

**Fig. 5:**
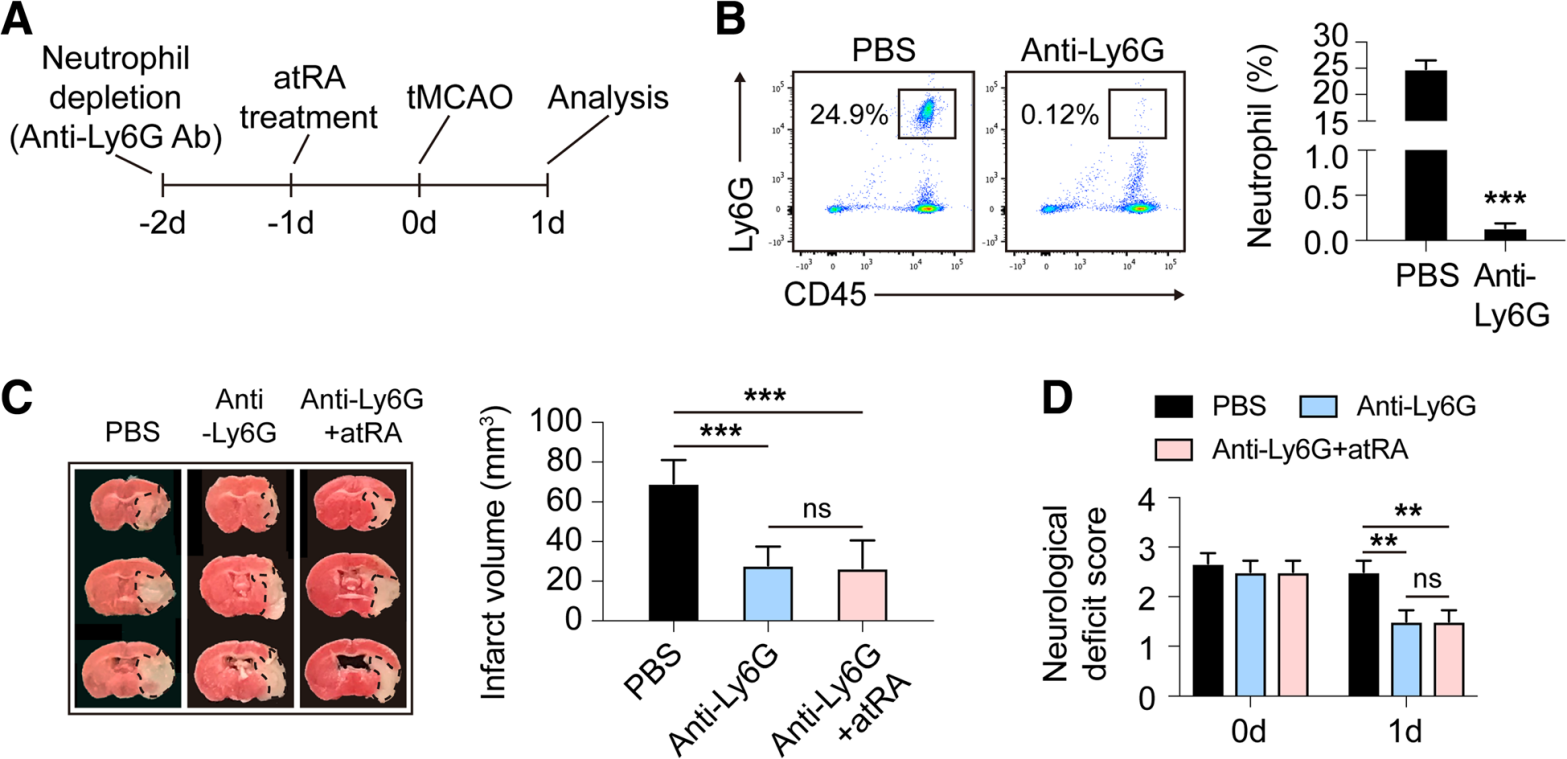
Neutrophil functions played vital role in the protective effects of atRA treatment. **a** Schematic diagram showing the time course of neutrophil depletion, atRA administration and tMCAO. **b** Frequency of neutrophil in peripheral blood was analyzed with flow cytometry to demonstrate the depletion efficacy of anti-Ly6G antibodies. Percentage of CD11b^+^CD45^hi^Ly6G^+^ neutrophil among singlets were displayed. *N* = 3 mice per group. ***P ≤ 0.001, versus PBS-treated group in *t* test. **c** Infarct volume of mice was quantified with TTC (red)-stained coronal sections (*N* = 6 mice per group). ***P ≤ 0.001 in one-way ANOVA. **d** Neurological deficit score was assessed right after reperfusion and 1d after tMCAO (*N* = 6 mice per group). **P ≤ 0.01 in two-way ANOVA

To examine the role of neutrophil in atRA-afforded protection in ischemic stroke, circulating neutrophils in male C57/BL6 wild-type (WT) mice were depleted with anti-Ly6G antibodies (50μg/mouse) 48 h before tMCAO (Fig. 5a). As described previously, elimination of the circulating neutrophil (Fig. 5b) protected against ischemic stroke [24–26] (Fig. 5c). Interestingly, atRA pre-treatment (administering atRA (1 mg/kg, i.p.) at 24 h before tMCAO and immediately after reperfusion) failed to further reduce infarct sizes (Fig. 5c) or neurological deficits (Fig. 5d) of the neutrophil-eliminated mice. These results indicate that the functions of neutrophil play an important role in the protective effects of atRA in ischemic stroke.

### All-trans retinoic acid downregulated STAT1 signaling in neutrophil

**Fig. 6:**
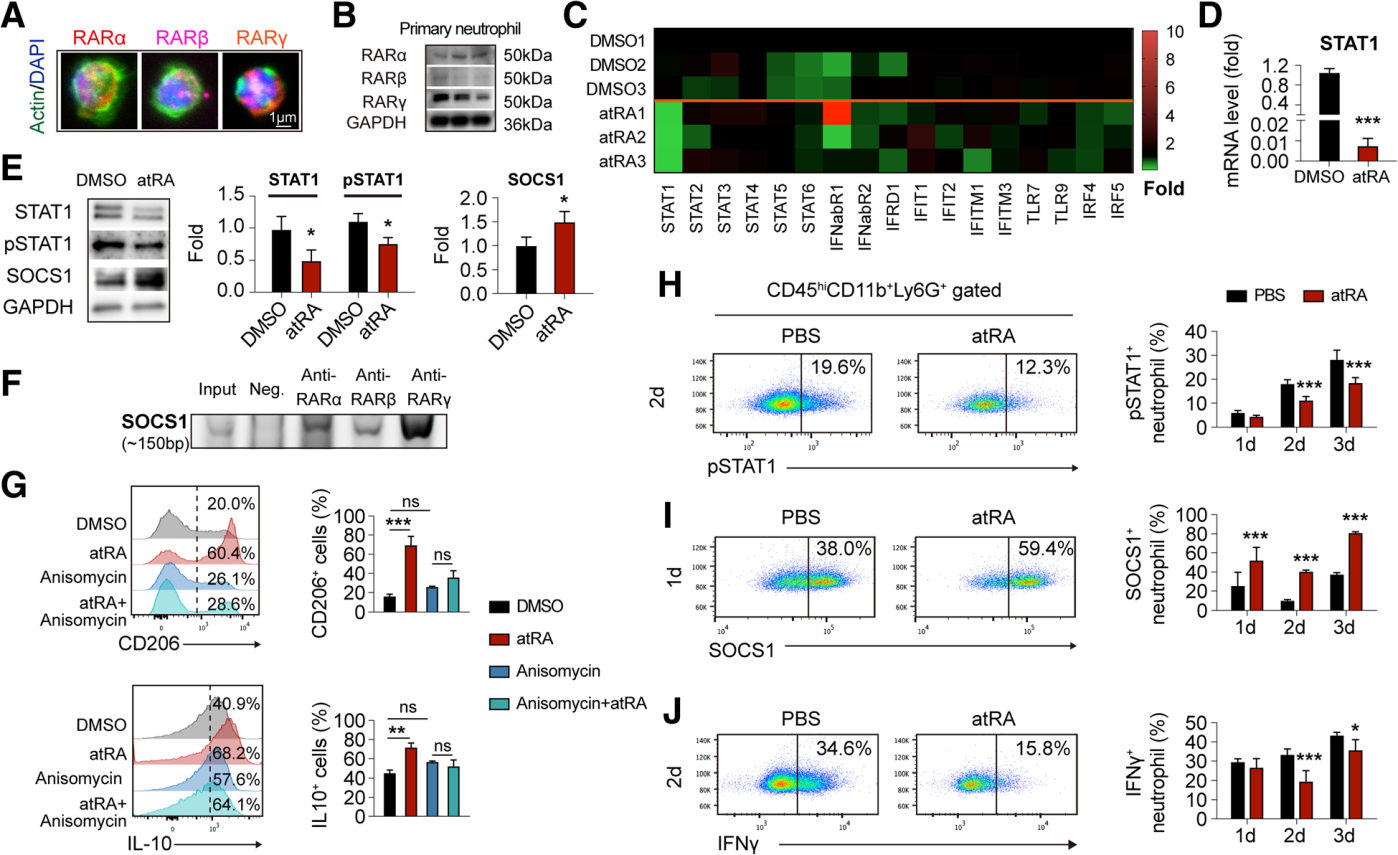
All-trans retinoic acid modified neutrophil functions through STAT1 signaling. **a**, **b** Expression of RAR-α, -β, and -γ in neutrophil was illustrated with immunofluorescent staining (**a**) and Western blot (**b**). **c** Heat map showing the modification of atRA treatment to STATs signaling in neutrophil. In the heat map, data are displayed as fold change to neutrophils from DMSO-treated group. Black, expression un-altered, fold change = 1; Red, expression up-regulated, fold change > 1; Green, expression downregulated, fold change < 1. **d** Quantification of the mRNA expression of STAT1 in neutrophil after atRA treatment. Quantification of markers that without significant difference between the two groups were displayed in Additional file 1: Figure S3. Experiments were repeated for 3 times. ***P ≤ 0.001, versus DMSO treated group in *t* test. **e** Protein expression of STAT1, phosphorylated STAT1 (pSTAT1, Y701), and SOCS1 in neutrophil after atRA treatment. Experiments were repeated for three times. *P ≤ 0.05, versus DMSO-treated group in *t* test. **f** Representative image of ChIP experiments showing that RARs could bind to SOCS1 gene. Experiments were repeated for three times. **g** Anisomycin (25 μg/ml) was treated to neutrophil to over-activate STAT1 signaling and abrogate the suppression of STAT1 phosphorylation by SOCS1. Neutrophil expression of N2 markers CD206 and IL-10 was assessed with flow cytometry. Experiments were repeated for three times. **P ≤ 0.01, ***P ≤ 0.001 in one-way ANOVA. **h**–**j** Activation status of STAT1 signaling in neutrophil in the ischemic brain was assessed with flow cytometry at 1–3 days after tMCAO. Neutrophil in the stroke lesion of atRA pre-treated mice displayed down-regulated STAT1 phosphorylation (**h**, starting at 2 days), upregulated SOCS1 expression (**i**, starting at 1 day), and decreased IFNγ expression (**j**, starting at 2 days). Representative flow panels of the expression of pSTAT1, SOCS1, and IFNγ were shown. Percentage among CD11b^+^CD45^hi^Ly6G^+^ neutrophil were calculated and displayed. *N* = 4 mice per group. *P ≤ 0.05, ***P ≤ 0.001, versus PBS-treated group in two-way ANOVA

All-trans retinoic acid is the ligand of retinoic acid receptors (RARs). As assessed with immunostaining and Western blot, we demonstrated that neutrophil expressed all the three types of RARs (namely RARα, RARβ and RARγ) though in different levels (Fig. 6a, b). Expressions of RARs by neutrophil provide a rationale that neutrophil could be modulated by atRA but the underlying mechanisms remain to be elusive. It has been established that signal transducers and activators of transcription (STAT) family plays an important role in neutrophil function [27]. Moreover, functions of STAT family are associated with the polarity of macrophage [28]. Therefore, the impact of atRA treatment on STATs signaling in neutrophils was analyzed. The mRNA expression of STATs and the relevant molecules in primary cultured neutrophil after atRA treatment was measured with qPCR (Fig. 6c, Additional file 1: Figure S4). Although the expression of most targets was stable, mRNA level of STAT1 was decreased after atRA treatment (Fig. 6d). As analyzed with Western blot, the protein expression of STAT1, as well as phosphorylated-STAT1 (pSTAT1, Y701), were downregulated by atRA treatment accordingly (Fig. 6e). We quantified the ratio of pSTAT1 to STAT1 and found that atRA did not alter the ratio of STAT1 phosphorylation (data not shown). It is known that phosphorylation and gene expression of STAT1 could be inhibited by suppressor of cytokine signaling 1 (SOCS1) [29, 30]. Therefore, protein level of SOCS1 in neutrophil after atRA treatment was assessed. Strikingly, atRA upregulated the protein expression of SOCS1 in neutrophil as measured with Western blot (Fig. 6e), which could downregulate expression of STAT1 protein and impede activation of STAT1 signaling.

It was documented that RARs could directly modulated the transcription of SOCS1 [31]. In primary cultured neutrophil, we found that all three types of RARs could bind to SOCS1 gene as assessed with ChIP experiments (Fig. 6f), which indicated that RARs participated in the regulation of gene transcription of SOCS1. However, modulation of STAT1 transcription by RARs was not observed (data not shown). When over-activating STAT1 signaling with anisomycin to confront the function of SOCS1 in neutrophil, atRA failed to induce N2 phenotype (Fig. 6g), which revealed the decisive role of SOCS1-STAT1 signaling in the effects of atRA on neutrophil polarity.

In compliance with the in vitro data, prophylactic atRA treatment inhibited STAT1 signaling in neutrophil at 1–3 days after stroke in vivo (Fig. 6h–j). In atRA pre-treated group, atRA (1  mg/kg, i.p.) was applied to mice at 24 h before tMCAO and immediately after reperfusion. Injection of atRA was repeated every 24 h until sacrifice. As assessed with flow cytometry, phosphorylated STAT1 was downregulated (Fig. 6h) while the expression of SOCS1 (Fig. 6i) was increased in neutrophil within the ischemic lesion of atRA pre-treated mice. Accordingly, expression of interferon gamma (IFNγ), as a down-stream target of STAT1 signaling, was decreased in neutrophil in atRA pre-treated mice (Fig. 6j). Consequently, we purpose that atRA modulates neutrophil functions through upregulating SOCS1 expression, which subsequently suppresses the expression and activation of STAT1 and the down-stream signaling.

### Post-stroke administration of atRA offered therapeutic protection against cerebral ischemia

**Fig. 7:**
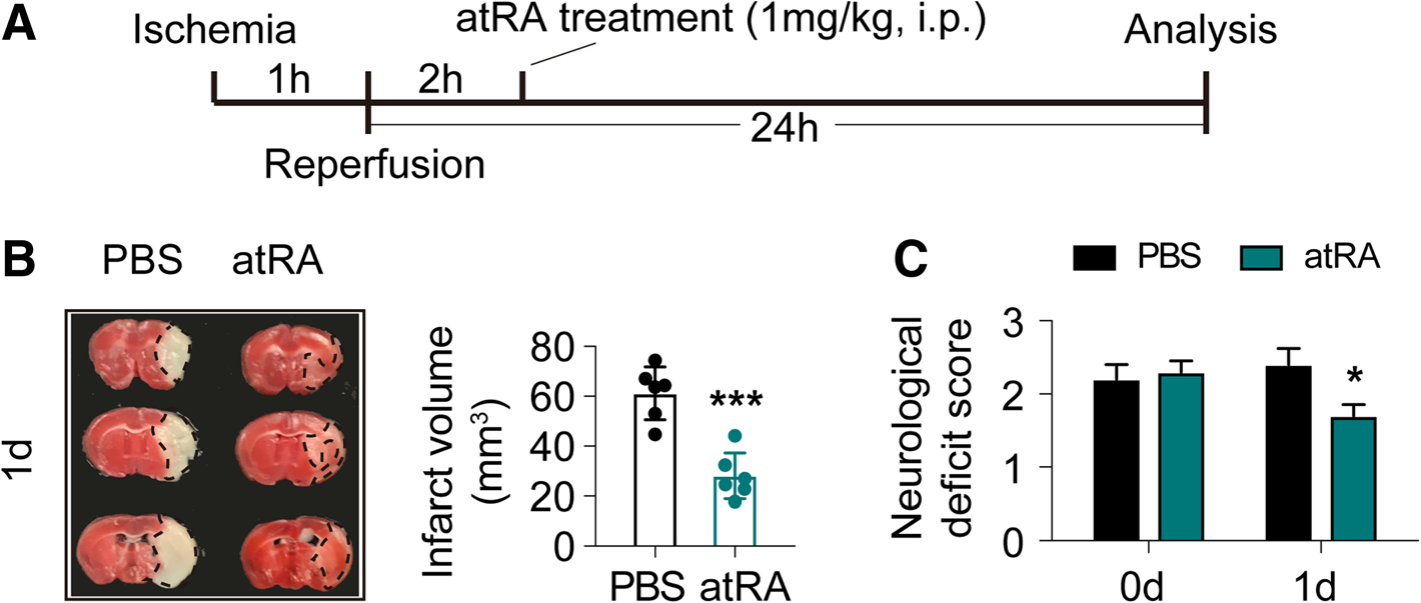
Post-stroke administration of atRA afforded efficient protection against cerebral ischemia. **a** Schematic diagram showing the time course of atRA treatment after ischemic stroke. **b** Infarct volume of mice was quantified with TTC (red)-stained coronal sections (*N* = 6 mice per group). ***P ≤ 0.001, versus PBS-treated group in *t* test. **c** Neurological deficit score was assessed right after reperfusion and 1d after tMCAO (*N* = 6 mice per group). *P ≤ 0.05, versus PBS-treated group in two-way ANOVA

**Fig. 8:**
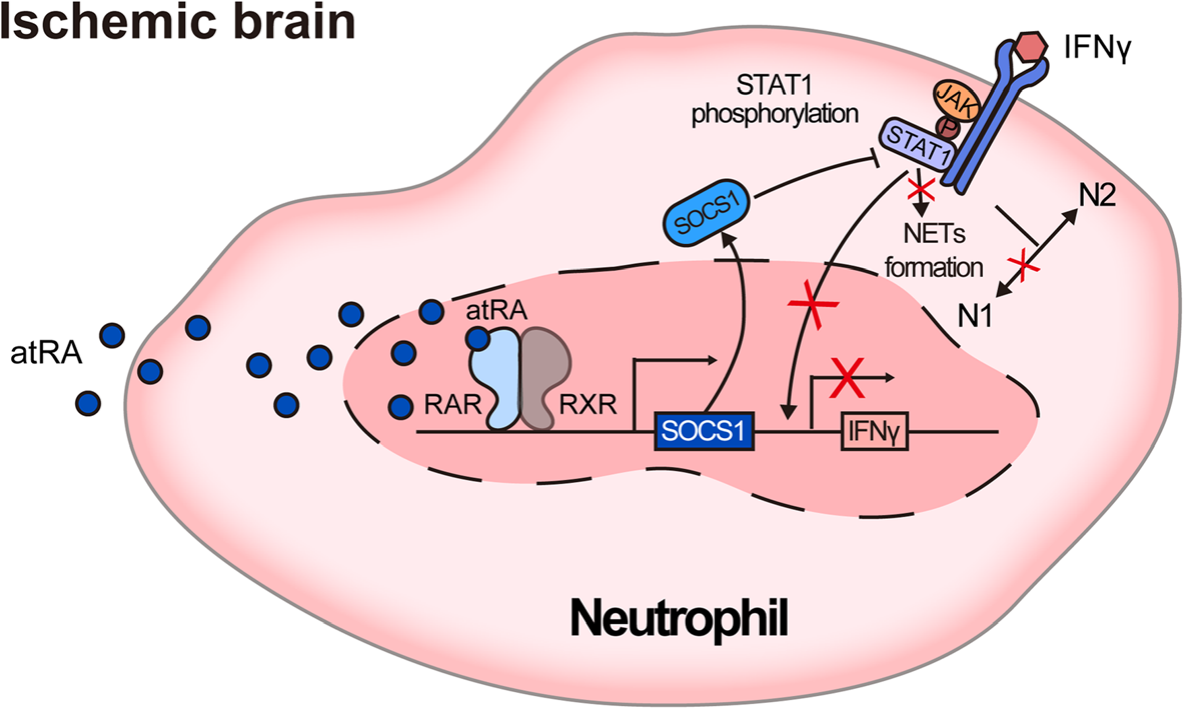
Schematic diagram displaying molecular mechanisms of the functional modification to neutrophil by atRA

In clinical practice, prophylactic treatment has had limited success in ischemic stroke. The most practical mode of management continues to be post-stroke systemic treatment. Therefore, to better establish the application value of atRA in ischemic stroke, we evaluated the therapeutic effects of atRA on an established murine stroke model tMCAO. Male C57BL/6 WT mice were subjected to tMCAO then treated with atRA (1 mg/kg, i.p.) or PBS (Veh) at 2 h after reperfusion (Fig. 7a). Stroke severity was assessed at 1 d after stroke. Similar to prophylactic treatment, mice accepted atRA therapy after stroke exhibited significantly decreased infarct volumes (Fig. 7b) and reduced neurological deficits (Fig. 7c). Moreover, we did not observe any difference in infarct volume between atRA pre-treated mice (Mean ± SEM = 21.22 ± 3.81) and those that had post-stroke atRA treatment (Mean ± SEM = 28.09 ± 3.75) (*P* = 0.25, *T* test). Conclusively, administration of atRA before or after stroke could both significantly protect against cerebral ischemia (Fig. 8).

## Discussion

Modulating post-stroke neural inflammation has been suggested as an important therapeutic strategy in cerebral ischemia [32]. All-trans retinoic acid is a well-established inflammatory modulator which has raised interest in the field of stroke therapy [9, 10, 33–35]. The current study demonstrates that atRA prophylactic treatment modulates post-stroke neural inflammation through regulation of neutrophil functions. Polarization of neutrophil was directed toward the beneficial N2 phenotype by atRA treatment, while the detrimental NETs formation was inhibited. The underlying mechanism was associated with the inhibition to STAT1 signaling by atRA treatment.

Although neutrophils are well-known to be involved in acute inflammation and injury [34] because of their lability. Our results illustrated that atRA treatment limited neutrophil counts in stroke lesion, suggesting that atRA is able to control stroke development in the early stage. The possible mechanisms could be attributed to decreased neutrophil entry and/or increased neutrophil clearance. A previous study by Kong L et al [33] demonstrated that atRA treatment could protect BBB after stroke, while we found that administration of atRA decreased neutrophil-attracting chemokines in the ischemic brain, which impeded neutrophil infiltration to stroke lesion. Nevertheless, whether atRA could prevent transmigration of neutrophil through brain endothelial cells still remains elusive, which is worth further study in the future. On the other hand, we showed that neutrophil clearance by microglia/macrophage was enhanced in atRA-treated mice, which could facilitate neutrophil retreat from stroke lesion. Collectively, atRA treatment impedes neutrophil infiltration and promotes neutrophil clearance, which synergistically restricts neutrophil accumulation in ischemic lesions. Previous study has illustrated that the reduction of neutrophil accumulation protected against ischemic stroke at least during the acute phase [36], which supports the point of view that the therapeutic effects of atRA is associated with decreased neutrophil accumulation in stroke lesion.

The evidence that reduced neutrophil count in stroke lesion signifies better stroke outcomes indicates that any approaches that reduced neutrophil accumulation could be beneficial, including preventing neutrophil entry as well as accelerating neutrophil clearance. It has been proved that N2 phenotype of neutrophil favors its clearance by macrophage and thus is protective. Our data indicated that atRA directs neutrophil polarization toward N2 phenotype. Therefore, atRA prevents neutrophil infiltration, and accelerates its clearance via inducing an N2 phenotype, which altogether reduces neutrophil accumulation, and offers a protective effect. When we eliminated neutrophil with anti-Ly6G antibodies, mechanisms relevant to neutrophil were dispelled. atRA fails to offer further protection in the absence of neutrophil, indicating that protective effects of atRA were at least partially relied on its role in suppressing neutrophil. Nevertheless, since the behavior of neutrophil is regulated by multiple players in stroke lesion [36]. Apparently, chemokines released by macrophage and other neural cells affect neutrophil infiltration into the ischemic brain, while phagocytosis of macrophage decided the pace of neutrophil retreat. Thus, though neutrophils play an important central role in the therapeutic process of atRA in stroke, other cells including macrophages could also participate in the cellular mechanisms of atRA treatment.

It is not surprising that atRA inhibited STAT1 signaling in neutrophil after ischemic stroke. STAT1 signaling is associated with M1 polarization in macrophages, and is antagonized with the effect of signal transducer and activator of transcription 6 (STAT6) signaling which directs macrophage toward M2 phenotype. Similar to macrophages, neutrophils display opposing properties, namely the pro-inflammatory N1 phenotype and the anti-inflammatory N2 phenotype. After atRA treatment, neutrophils, in which STAT1 signaling was inhibited, polarized toward N2 phenotype. Therefore, we infer that atRA downregulates STAT1 signaling, thus facilitates N2 polarization of neutrophil. We observed that expression of N1 markers in neutrophil was hardly affected by atRA treatment. One explanation could be that although the N2 property of neutrophils increased after atRA treatment, activation status of neutrophils remained un-altered.

Since it is known that activation of STAT1 signaling promotes NETs formation [37], which may cause further neural injury after the primary ischemic attack, we explored the role of atRA treatment in NETs and showed that atRA significantly inhibited NETs formation after ischemic stroke, which might be associated with the suppression of STAT1 signaling by atRA. It is unclear whether atRA directly inhibits NETs or inhibits STAT1 signaling and then indirectly suppresses NETs.

The short life and lability of the neutrophil create obstacles for the measurement of neutrophil functions by gene editing techniques, which in turn have impeded research in this area. Up to date, the molecular mechanisms of neutrophil polarization and NETs formation continue to be elusive. Our findings illustrating that STAT1 signaling participates in the regulation of neutrophil polarity and NETs formation may be helpful. Nevertheless, further experiments with transgenic mice to provide direct evidence are in need.

In the current study, we documented significant reduced infarct volume in mice with atRA treatment (1 mg/kg atRA i.p. at 2 h after 60 min of tMCAO). Nevertheless, in study by Sato et al., when mice were treated with atRA for 10 mg/kg, i.p. at 1 h after 60 min of tMCAO, the authors observed reduced infarct volume in mice treated with atRA though the protection was not significant [13]. Of note, reproducibility between labs is a major translational obstacle worldwide which may be attributed to multiple interfering factors. For example, microbiota differences between commercial breeders may affect post-stroke immune response. With various microbiota, promising therapy proposed by one lab may showed modest protection in the other [38]. The divert results in the two experiments could be the result of similar reasons.

## Conclusions

In conclusion, our study elucidates that atRA modulated post-stroke neural inflammation and regulated neutrophil functions, favoring the beneficial N2 phenotype and impeding the formation of NETs. The underlying mechanisms are associated with STAT1 signaling. With its protective potency in ischemic stroke, further clinical investigation into atRA as a promising therapeutic agent in patients with ischemic stroke is warranted.

## Additional file

Additional file 1: **Table S1.** Summary of Experimental Groups and Mortality Rate of Mice (C57BL/6). **Table S2.** Primers used in the study. **Figure S1.** Comparison of undisturbed brains of atRA- and PBS- pre-treated mice. **Figure S2.** Comparison of mRNA expression of inflammatory mediators in ischemic brains between atRA- and PBS- pre-treated mice. **Figure S3.** Comparison of mRNA expression of phenotypic markers in neutrophils isolated from ipsilateral brains of atRA- and PBS-treated mice. **Figure S4.** Modification of atRA treatment to STATs signaling in neutrophil. (DOCX 2879 kb)

## Data Availability

The datasets used and / or analyzed during the current study are available from the corresponding author on reasonable request.

## Abbreviations

Arg1: Arginase 1
BAFF: B cell activating factor
CCL1: Chemokine (C-C motif) ligand 1
CCL3: Chemokine (C-C motif) ligand 3
CCL5: Chemokine (C-C motif) ligand 5
CCL7: Chemokine (C-C motif) ligand 7
CTX: Cortex
CXCL1: Chemokine (C-X-C motif) ligand 1
CXCL10: Chemokine (C-X-C motif) ligand 10
CXCL11: Chemokine (C-X-C motif) ligand 11
CXCL2: Chemokine (C-X-C motif) ligand 2
CXCL3: Chemokine (C-X-C motif) ligand 3
CXCL5: Chemokine (C-X-C motif) ligand 5
CXCL7: Chemokine (C-X-C motif) ligand 7
CXCL9: Chemokine (C-X-C motif) ligand 9
DMSO: Dimethyl sulfoxide
GAPDH: Glyceraldehyde 3-phosphate dehydrogenase
IFIT1: Interferon-induced protein with tetratricopeptide repeats 1
IFIT2: Interferon-induced protein with tetratricopeptide repeats 2
IFITM1: Interferon-induced transmembrane protein 1
IFITM3: Interferon-induced transmembrane protein 3
IFNabR1: Interferon alpha/beta receptor 1
IFNabR2: Interferon alpha/beta receptor 2
IFNγ: Interferon gamma
IFRD1: Interferon-related developmental regulator 1
IL-10: Interleukin 10
IL-12a: Interleukin 12 alpha
IL-17a: Interleukin 17 alpha
IL-1a: Interleukin 1 alpha
IL-21: Interleukin 21
IL-6: Interleukin 6
IRF4: Interferon regulatory factor 4
IRF5: Interferon regulatory factor 5
MMP10: Matrix metalloproteinase-10
MMP3: Matrix metalloproteinase-3
MMP8: Matrix metalloproteinase-8
MMP9: Matrix metalloproteinase-9
MPO: Myeloperoxidase
NE: Norepinephrine
NLRP3: NACHT, LRR, and PYD domains-containing protein 3
PAD4: Peptidyl arginine deiminase 4
pSTAT1: Phosphorylated signal transducer and activator of transcription 1
RARα: Retinoic acid receptor alpha
RARβ: Retinoic acid receptor beta
RARγ: Retinoic acid receptor gamma
SOCS1: Suppressor of cytokine signaling 1
STAT1: Signal transducer and activator of transcription 1
STAT2: Signal transducer and activator of transcription 2
STAT3: Signal transducer and activator of transcription 3
STAT4: Signal transducer and activator of transcription 4
STAT5: Signal transducer and activator of transcription 5
STAT6: Signal transducer and activator of transcription 6
STR: Striatum
TGFβ: Transforming growth factor beta
TLR7: Toll-like receptor 7
TLR9: Toll-like receptor 9
TMP2: Transmembrane protein 3
TMP3: Transmembrane protein 2
TNFα: Tumor necrosis factor alpha
VEGF: Vascular endothelial growth factor

## References

[1] Cuartero MI, Ballesteros I, Moraga A, Nombela F, Vivancos J, Hamilton JA (2013). N2 neutrophils, novel players in brain inflammation after stroke: modulation by the PPARgamma agonist rosiglitazone. Stroke..

[2] Ducroux C, Di Meglio L, Loyau S, Delbosc S, Boisseau W, Deschildre C (2018). Thrombus neutrophil extracellular traps content impair tPA-induced thrombolysis in acute ischemic stroke. Stroke..

[3] Liu ZM, Wang KP, Ma J, Guo Zheng S (2015). The role of all-trans retinoic acid in the biology of Foxp3+ regulatory T cells. Cell Mol Immunol.

[4] Lu L, Lan Q, Li Z, Zhou X, Gu J, Li Q (2014). Critical role of all-trans retinoic acid in stabilizing human natural regulatory T cells under inflammatory conditions. Proc Natl Acad Sci U S A.

[5] Ma J, Liu Y, Li Y, Gu J, Liu J, Tang J (2014). Differential role of all-trans retinoic acid in promoting the development of CD4+ and CD8+ regulatory T cells. J Leukoc Biol.

[6] Lu L, Ma J, Li Z, Lan Q, Chen M, Liu Y (2011). All-trans retinoic acid promotes TGF-beta-induced Tregs via histone modification but not DNA demethylation on Foxp3 gene locus. PLoS One.

[7] Lu L, Zhou X, Wang J, Zheng SG, Horwitz DA (2010). Characterization of protective human CD4CD25 FOXP3 regulatory T cells generated with IL-2, TGF-beta and retinoic acid. PLoS One.

[8] Zhou X, Kong N, Wang J, Fan H, Zou H, Horwitz D (2010). Cutting edge: all-trans retinoic acid sustains the stability and function of natural regulatory T cells in an inflammatory milieu. J Immunol.

[9] Li Y, Gao X, Wang Q, Yang Y, Liu H, Zhang B (2017). Retinoic acid protects from experimental cerebral infarction by upregulating GAP-43 expression. Braz J Med Biol Res.

[10] Sabbaghziarani F, Mortezaee K, Akbari M, Kashani IR, Soleimani M, Moini A (2017). Retinoic acid-pretreated Wharton's jelly mesenchymal stem cells in combination with triiodothyronine improve expression of neurotrophic factors in the subventricular zone of the rat ischemic brain injury. Metab Brain Dis.

[11] Nally JE, Clayton RA, Wakelam MJ, Thomson NC, McGrath JC (1994). Angiotensin II enhances responses to endothelin-1 in bovine bronchial smooth muscle. Pulm Pharmacol.

[12] Stetler RA, Cao G, Gao Y, Zhang F, Wang S, Weng Z (2008). Hsp27 protects against ischemic brain injury via attenuation of a novel stress-response cascade upstream of mitochondrial cell death signaling. J Neurosci.

[13] Sato Y, Meller R, Yang T, Taki W, Simon RP (2008). Stereo-selective neuroprotection against stroke with vitamin A derivatives. Brain Res.

[14] Yang Y, Liu H, Zhang H, Ye Q, Wang J, Yang B (2017). ST2/IL-33-dependent microglial response limits acute ischemic brain injury. J Neurosci.

[15] Adelson JD, Barreto GE, Xu L, Kim T, Brott BK, Ouyang YB (2012). Neuroprotection from stroke in the absence of MHCI or PirB. Neuron..

[16] Chen Z, Barbi J, Bu S, Yang HY, Li Z, Gao Y (2013). The ubiquitin ligase Stub1 negatively modulates regulatory T cell suppressive activity by promoting degradation of the transcription factor Foxp3. Immunity..

[17] Gao Y, Tang J, Chen W, Li Q, Nie J, Lin F (2015). Inflammation negatively regulates FOXP3 and regulatory T-cell function via DBC1. Proc Natl Acad Sci U S A.

[18] Griffith JW, Sokol CL, Luster AD (2014). Chemokines and chemokine receptors: positioning cells for host defense and immunity. Annu Rev Immunol.

[19] Gelderblom M, Leypoldt F, Steinbach K, Behrens D, Choe CU, Siler DA (2009). Temporal and spatial dynamics of cerebral immune cell accumulation in stroke. Stroke..

[20] Shaul ME, Levy L, Sun J, Mishalian I, Singhal S, Kapoor V (2016). Tumor-associated neutrophils display a distinct N1 profile following TGFbeta modulation: a transcriptomics analysis of pro- vs. antitumor TANs. Oncoimmunology..

[21] Lood C, Blanco LP, Purmalek MM, Carmona-Rivera C, De Ravin SS, Smith CK (2016). Neutrophil extracellular traps enriched in oxidized mitochondrial DNA are interferogenic and contribute to lupus-like disease. Nat Med.

[22] Zawrotniak M, Rapala-Kozik M (2013). Neutrophil extracellular traps (NETs)—formation and implications. Acta Biochim Pol.

[23] Darrah E, Andrade F (2012). NETs: the missing link between cell death and systemic autoimmune diseases?. Front Immunol.

[24] Li P, Mao L, Liu X, Gan Y, Zheng J, Thomson AW (2014). Essential role of program death 1-ligand 1 in regulatory T-cell-afforded protection against blood-brain barrier damage after stroke. Stroke..

[25] Li P, Mao L, Zhou G, Leak RK, Sun BL, Chen J (2013). Adoptive regulatory T-cell therapy preserves systemic immune homeostasis after cerebral ischemia. Stroke..

[26] Li P, Gan Y, Sun BL, Zhang F, Lu B, Gao Y (2013). Adoptive regulatory T-cell therapy protects against cerebral ischemia. Ann Neurol.

[27] Martinelli S, Urosevic M, Daryadel A, Oberholzer PA, Baumann C, Fey MF (2004). Induction of genes mediating interferon-dependent extracellular trap formation during neutrophil differentiation. J Biol Chem.

[28] Nguyen VT, Benveniste EN (2000). Involvement of STAT-1 and ets family members in interferon-gamma induction of CD40 transcription in microglia/macrophages. J Biol Chem.

[29] Bai J, Wu L, Chen X, Wang L, Li Q, Zhang Y (2018). Suppressor of cytokine signaling-1/STAT1 regulates renal inflammation in mesangial proliferative glomerulonephritis models. Front Immunol.

[30] Liang YB, Tang H, Chen ZB, Zeng LJ, Wu JG, Yang W (2017). Downregulated SOCS1 expression activates the JAK1/STAT1 pathway and promotes polarization of macrophages into M1 type. Mol Med Rep.

[31] Liu S, Ren S, Howell P, Fodstad O, Riker AI (2008). Identification of novel epigenetically modified genes in human melanoma via promoter methylation gene profiling. Pigment Cell Melanoma Res.

[32] Cai W, Liu S, Hu M, Sun X, Qiu W, Zheng S (2018). Post-stroke DHA treatment protects against acute ischemic brain injury by skewing macrophage polarity toward the M2 phenotype. Transl Stroke Res.

[33] Kong L, Wang Y, Wang XJ, Wang XT, Zhao Y, Wang LM (2015). Retinoic acid ameliorates blood-brain barrier disruption following ischemic stroke in rats. Pharmacol Res.

[34] Tan Z, Jiang R, Wang X, Wang Y, Lu L, Liu Q (2013). RORgammat+IL-17+ neutrophils play a critical role in hepatic ischemia-reperfusion injury. J Mol Cell Biol.

[35] Huang Zhiyi, Liu Yu, Qi Guangying, Brand David, Zheng Song (2018). Role of Vitamin A in the Immune System. Journal of Clinical Medicine.

[36] Herz J, Sabellek P, Lane TE, Gunzer M, Hermann DM, Doeppner TR (2015). Role of neutrophils in exacerbation of brain injury after focal cerebral ischemia in Hyperlipidemic mice. Stroke..

[37] Gul E, Sayar EH, Gungor B, Eroglu FK, Surucu N, Keles S (2018). Type I IFN-related NETosis in ataxia telangiectasia and Artemis deficiency. J Allergy Clin Immunol.

[38] Sadler R, Singh V, Benakis C, Garzetti D, Brea D, Stecher B (2017). Microbiota differences between commercial breeders impacts the post-stroke immune response. Brain Behav Immun.

