# Supplementary material for: All trans-retinoic acid protects against acute ischemic stroke by modulating neutrophil functions through STAT1 signaling
Source: J Neuroinflammation. 2019 Aug 31;16:175. doi: 10.1186/s12974-019-1557-6 (PMC6717357; doi:10.1186/s12974-019-1557-6)
Supplement: Supplementary file 1 — Table S1. Summary of Experimental Groups and Mortality Rate of Mice (C57BL/6). Table S2. Primers used in the study. Figure S1. Comparison of undisturbed brains of atRA- and PBS- pre-treated mice. Figure S2. Comparison of mRNA expression of inflammatory mediators in ischemic brains between atRA- and PBS- pre-treated mice. Figure S3. Comparison of mRNA expression of phenotypic markers in neutrophils isolated from ipsilateral brains of atRA- and PBS-treated mice. Figure S4. Modification of atRA treatment to STATs signaling in neutrophil. (DOCX 2879 kb) [file 12974_2019_1557_MOESM1_ESM.docx]

**Table S1. Summary of Experimental Groups and Mortality Rate of Mice (C57BL/6).**

| **Experimental Groups** | **Infarct Volume Analysis** | **Neurological Deficit Score** | **Immuno-**  **fluorescence Staining** | **FACS** | **RT-PCR** | **Exclusion** | **Mortality** | **Subtotal** |
| --- | --- | --- | --- | --- | --- | --- | --- | --- |
| **Result 1** |  |  |  |  |  |  |  |  |
| PBS | 11 | 11 | 6 |  |  | 1 (5.6%) | 1 (5.6%) | 18 |
| atRA | 10 | 10 | 6 |  |  | 2 (11.1%) | 2 (11.1%) | 18 |
| **Result 2** |  |  |  |  |  |  |  |  |
| PBS |  |  |  | 6 | 5 | 2 (15.4%) | 2 (15.4%) | 13 |
| atRA |  |  |  | 6 | 5 | 0 | 0 | 11 |
| **Result 3** |  |  |  |  |  |  |  |  |
| PBS |  |  | 4 | 16 | 8 | 1 (3.6%) | 1 (3.6%) | 28 |
| atRA |  |  | 4 | 17 | 8 | 0 | 0 | 28 |
| **Result 4** |  |  |  |  |  |  |  |  |
| PBS |  |  | 4* |  |  | 0 | 0 | 4* |
| atRA |  |  | 4* |  |  | 0 | 0 | 4* |
| In vitro experiments |  |  |  |  |  |  |  | 12 |
| **Result 5** |  |  |  |  |  |  |  |  |
| PBS | 6 | 6 |  | 3^ |  | 0 | 0 | 6 |
| Anti-Ly6G | 6 | 6 |  | 3^ |  | 0 | 0 | 6 |
| Anti-Ly6G+atRA | 6 | 6 |  |  |  | 0 | 0 | 6 |
| **Result 6** |  |  |  |  |  |  |  |  |
| PBS |  |  |  | 12 |  | 2 (14.3) | 2 (14.3) | 14 |
| atRA |  |  |  | 12 |  | 2 (14.3) | 1 (7.1) | 14 |
| In vitro experiments |  |  |  |  |  |  |  | 21 |
| **Result 7** |  |  |  |  |  |  |  |  |
| PBS | 6 | 6 |  |  |  | 0 | 0 | 6 |
| atRA | 6 | 6 |  |  |  | 0 | 0 | 6 |
| **Total** | 51 | 51 | 20 | 69 | 26 | 10 (4.8%) | 9 (4.3%) | 207 |

Exclusion included mice that with neurological deficit score < 1 or died after surgery. *Same sample used in Result 3. ^Blood sample from mice used in infarct volume analysis in Result 5.

**Table S2. Primers used in the study**

|  | Forward primer | Reverse primer |
| --- | --- | --- |
| CCL1 | AGTTCTTGGCTCCACCAGAC | CATCCTGTATCCACACGGCA |
| CCL3 | TGCCAAGTAGCCACATCGAG | GAGATGGGGGTTGAGGAACG |
| CCL5 | AAGTGTGTGCCAACCCAGAG | CCCATTTTCCCAGGACCGAG |
| CCL17 | GACCTTCCCGCTGAGGCATTTG | TCTGCTCTGTGGCTGCTCTTGG |
| CXCL1 | TGGCTGGGATTCACCTCAAG | CCGTTACTTGGGGACACCTT |
| CXCL2 | CATAGCCACTCTCAAGGGCG | AGGTACGATCCAGGCTTCCC |
| CXCL3 | CATCCAGAGCTTGACGGTGA | ACACATCCAGACACCGTTGG |
| CXCL5 | CCCCTTCCTCAGTCATAGCC | CTTCCACCGTAGGGCACTG |
| CXCL7 | CCTGGCGTCAAGAGAATCGT | CTTGGCTTGCCCGTCTTCAT |
| CXCL9 | GGGCAGAAGTTCCGTCTTGA | TACCGAAGGGAGGTGGACAA |
| CXCL10 | CTAGCTCAGGCTCGTCAGTT | CCCTTGGGAAGATGGTGGTTA |
| CXCL11 | GATCCAAGCAAGCTCGCCTCAT | ACGTGGCTGCATGTTCCAAGAC |
| TNFα | AGAAGTTCCCAAATGGCCTC | CCACTTGGTGGTTTGCTACG |
| IFNγ | ATGAACGCTACACACTGCATC | CCATCCTTTTGCCAGTTCCTC |
| IL-1a | AAGACAAGCCTGTGTTGCTGAAGG | TCCCAGAAGAAAATGAGGTCGGTC |
| IL-6 | TCCTACCCCAACTTCCAATGCTC | TTGGATGGTCTTGGTCCTTAGCC |
| IL-12a | CCTTGCATCTGGCGTCTACA | GTCTTCAGCAGGTTTCGGGA |
| IL-17a | TCCCTCTGTGATCTGGGAAG | CTCGACCCTGAAAGTGAAGG |
| IL-21 | TCAAGCCATCAAACCCTGGA | TGAATCATCTTTTGAAGGAGCCA |
| IL-10 | CCAAGCCTTATCGGAAATGA | TTTTCACAGGGGAGAAATCG |
| TGFβ | TGCGCTTGCAGAGATTAAAA | CGTCAAAAGACAGCCACTCA |
| Arg1 | TCACCTGAGCTTTGATGTCG | CTGAAAGGAGCCCTGTCTTG |
| MPO | GAACAATCAGTACCGGCCCA | TCTGGCGATTCAGTTTGGCT |
| NE | GGCAGGCATCTGCTTCGTAA | CCATGTCAGAGGCTGGATCTC |
| BAFF | TACCGAGGTTCAGCAACACC | TTCGTATAGTCGGCGTGTCG |
| MMP3 | CCCTGGGACTCTACCACTCA | AGTCCTGAGAGATTTGCGCC |
| MMP8 | CCACACACAGCTTGCCAATG | GCTTCTCTGCAACCATCGTG |
| MMP9 | CCAGCCGACTTTTGTGGTCT | TGGCCTTTAGTGTCTGGCTG |
| MMP10 | ATGGACACTTGCACCCTCAG | GGTGGAAGTTAGCTGGGCTT |
| TIMP3 | CTTTGTGGAGAGGTGGGACC | ATGCAGGCGTAGTGTTTGGA |
| TIMP2 | TTTCTAGCCACACCAGGCAG | GCATGACGGGAGTAAGGGAG |
| NLRP3 | ACGAGTCCTGGTGACTTTGTAT | CTTTCTCGGGCGGGTAATCTT |
| PAD4 | GGATGGTTGGGCTTCCACAG | TCCAATGTGCTTTGCGGAGG |
| CD206 | CAAGGAAGGTTGGCATTTGT | CCTTTCAGTCCTTTGCAAGC |
| VEGF | CGATTGAGACCCTGGTGGAC | GCTGGCTTTGGTGAGGTTTG |
| STAT1 | GCGAAGAGCGACCAAAAACA | CTGCAACAATGGTGAACCACG |
| STAT2 | TTGGGACTTCGGCTTCTTGACT | TGTCCGTCTGCAGCTTCATC |
| STAT3 | GGAGACAGTCGAGACCCCTG | CAGCTCCATGGGGAACGTGT |
| STAT4 | CTGAATGACGGTGCAAACGG | GTGGCACCAAGTGAGAAAGAG |
| STAT5 | TTGAGGGGAACTCTTCGGGA | TCTAAACAGGCGCAGGAAGG |
| STAT6 | CCCGGTCTCACCTAACTATGC | GTAGGTCTGTCCAGCGGTTC |
| IFNabR1 | GGTCATTACTGTCACCGCCA | ACACAGTACACAGTCAGCGG |
| IFNabR2 | GGTGTAGGGTCCTCCCATCT | TCAGAGGGCAGAGAAAGGGT |
| IFRD1 | CCCGCGCTAGAGAGAAACAT | GGCTAACAAGAGAGGCGAGG |
| IFIT1 | TGAGGAAGGCTGTCCGGTTA | TGGCTGCATAGCGAATGACA |
| IFIT2 | AGATGAGACTTAGAGGTGCTGC | TGTGTCAAAGCGCTCAAAGC |
| IFITM1 | TGCCCACACCCTAATGCTTC | CCCAGGCAGCAGAAGTTCAT |
| IFITM3 | AGCCTATGCCTACTCCGTGA | AGTGTGAAGGTTTTGAGCGTT |
| TLR7 | CAAAGCACGCAGCTCAAAGG | GGGAGCCAAGGACATCTTTCT |
| TLR9 | CTCCAACCGTATCCACCACC | GAGAAGTGCAGGGGGCTAAG |
| IRF4 | GGATTGTTCCAGAGGGAGCC | AGTTATGAACCTGCTGGGCTG |
| IRF5 | GGGCGCACAGCAAACAGA | CACTGAAGCCCTGGGTACTG |


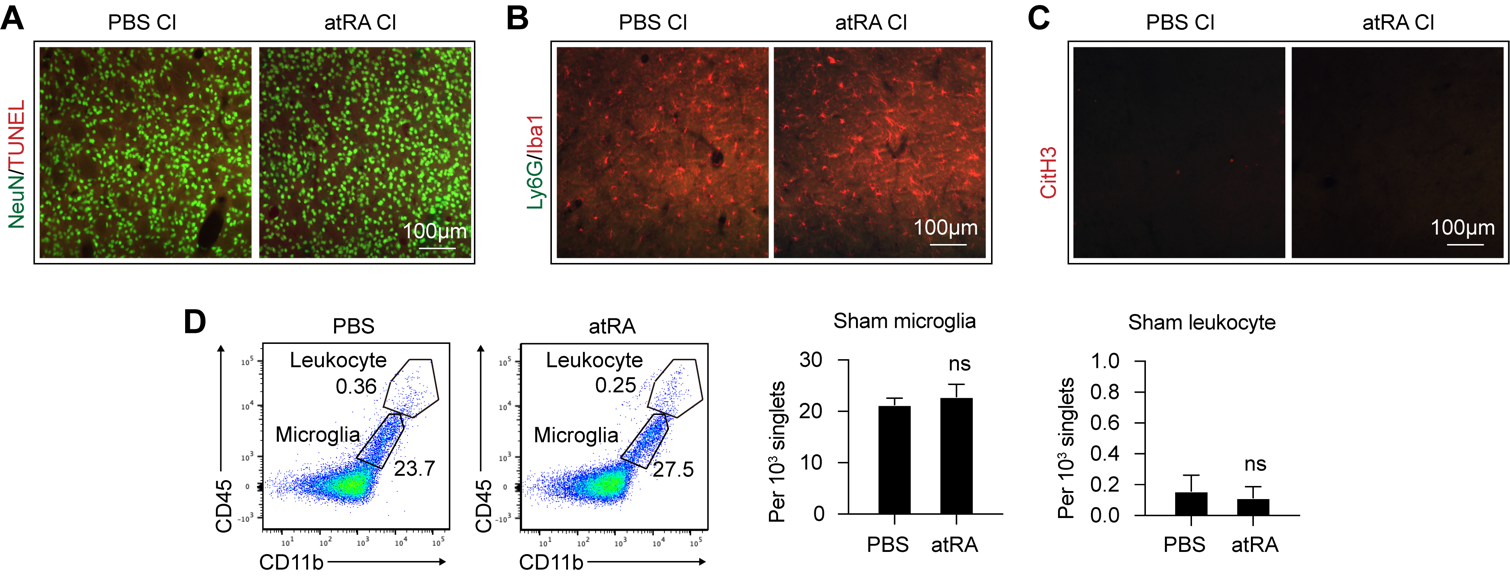


**Figure S1. Comparison of undisturbed brains of atRA- and PBS- pretreated mice. (A-C)** Comparison of contralateral brains from PBS- or atRA-pretreated mice. C57BL/6 mice were treated with atRA (1mg/kg, i.p.) or PBS 24h before 60min of cerebral ischemia. The treatment was repeated immediately after reperfusion. Mice were sacrificed at 1d after tMCAO. **(A)** Representative images showing TUNEL (green) co-labeling with NeuN (red) in contralateral brains of PBS- or atRA- pretreated mice at 1d after tMCAO. **(B)** Representative images showing Ly6G (green) co-labeling with Iba1 (red) in contralateral brains of PBS- or atRA- pretreated mice at 1d after tMCAO. **(C)** Representative images showing labeling of CitH3 (red) in contralateral brains of PBS- or atRA- pretreated mice at 1d after tMCAO. **(D)** Representative flow cytometric plots showing microglia (CD11b^+^CD45^int^) and infiltrated leukocyte (CD11b^+^CD45^hi^) in Sham operated brains from PBS- or atRA-pretreated mice and corresponding statistics. C57BL/6 mice were treated with atRA (1mg/kg, i.p.) or PBS 24h before Sham operation which was a same procedure as tMCAO except for no filament insertion. Mice were sacrificed at 1d after Sham operation. *N* = 3 in PBS- and atRA- pretreated groups. ns, non significance, compared with PBS-pretreated group in *t-test*.

**
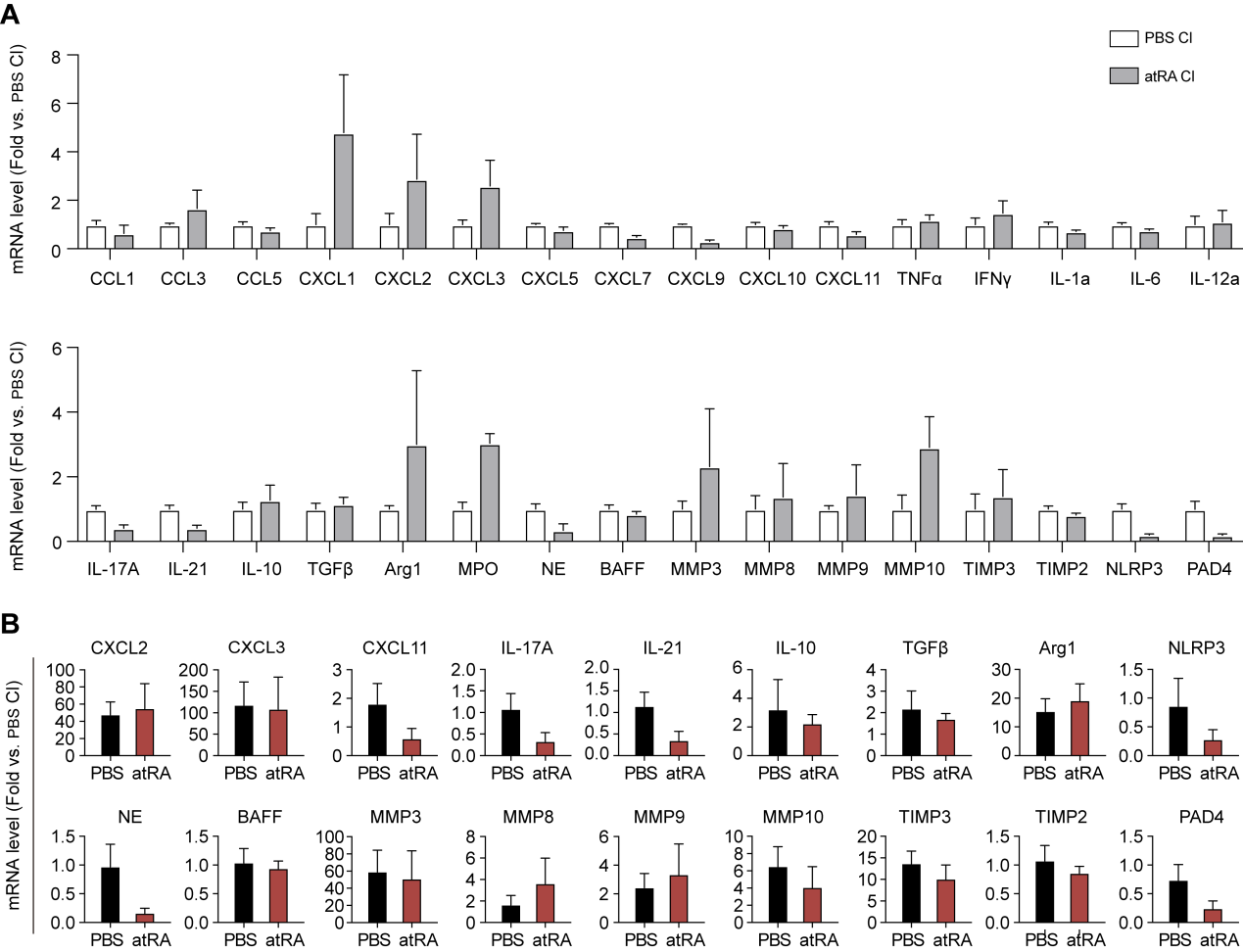
**

**Figure S2. Comparison of mRNA expression of inflammatory mediators in ischemic brains between atRA- and PBS- pretreated mice. (A)** Quantification of mRNA expression of inflammatory mediators in the contralateral brains between atRA- and PBS-treated mice as assessed with qPCR. mRNA expression was normalized to the level of the contralateral brain from PBS pre-treated mice. *N* = 3 in PBS Cl and atRA Cl groups. No significance difference between the two groups was observed. **(B)** Quantification of inflammatory markers without significant difference or fold change < 2 of mRNA expression in the ipsilateral brains between atRA- and PBS-treated mice as assessed with qPCR. mRNA expression was normalized to the level of the contralateral brain from PBS pre-treated mice. *N* = 5 in PBS Ip and atRA Ip groups. Quantification of markers that with significant change between PBS Ip and atRA Ip groups was displayed in **Figure 2**.


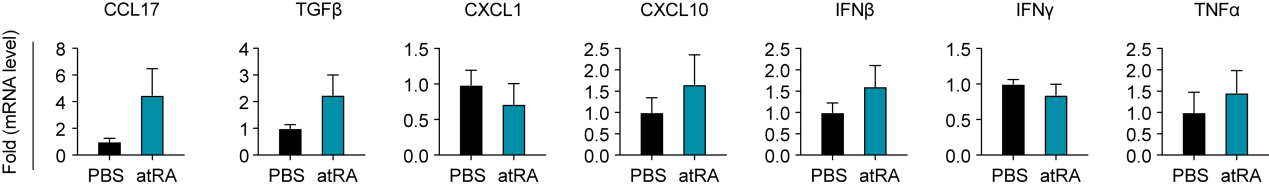


**Figure S3. Comparison of mRNA expression of phenotypic markers in neutrophils isolated from ipsilateral brains of atRA- and PBS-treated mice.** Expression of phenotypic markers in neutrophils isolated from the ipsilateral brains of atRA- and PBS-pretreated mice was assessed with qPCR (**Figure 3**). Quantification of markers that without signifcant difference in mRNA expression between the two groups was displayed. *N* = 8 mice per group.


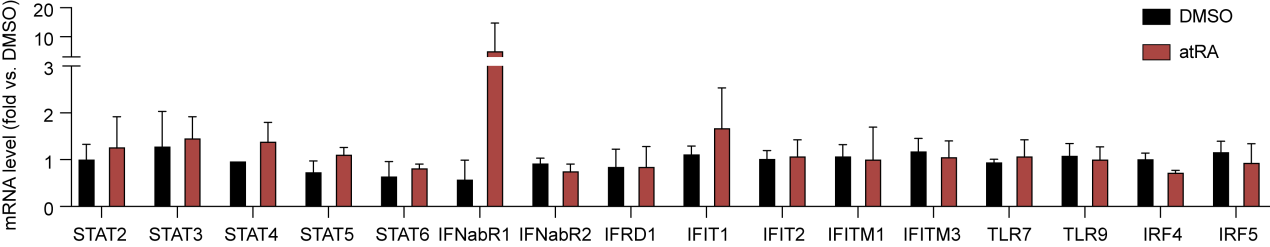


**Figure S4. Modification of atRA treatment to STATs signaling in neutrophil.** Quantification of mRNA expression of STATs and the relevant molecules in primary cultured neutrophil after atRA treatment was measured with qPCR (See **Figure 6**). Markers that without signicant difference between DMSO- and atRA-treated primary cultured neutrophils are displayed.
